# A multifunctional AIE gold cluster-based theranostic system: tumor-targeted imaging and Fenton reaction-assisted enhanced radiotherapy

**DOI:** 10.1186/s12951-021-01191-x

**Published:** 2021-12-20

**Authors:** Yue Hua, Yuan Wang, Xue Kang, Fan Xu, Zhen Han, Chong Zhang, Zhao-Yang Wang, Jun-Qi Liu, Xueli Zhao, Xiaoyuan Chen, Shuang-Quan Zang

**Affiliations:** 1 Henan Key Laboratory of Crystalline Molecular Functional Materials, Henan International Joint Laboratory of Tumor Theranostical Cluster Materials, Green Catalysis Center, and College of Chemistry, Zhengzhou University, Zhengzhou, 450001 China; 2 The First Affiliated Hospital of Zhengzhou University, Zhengzhou, 450000 China; 3 Departments of Diagnostic Radiology, Chemical and Biomolecular Engineering, and Biomedical Engineering, National University of Singapore, Singapore, 117545 Singapore; 4 Clinical Imaging Research Centre, Centre for Translational Medicine, Yong Loo Lin School of Medicine, National University of Singapore, Singapore, 117599 Singapore; 5 Nanomedicine Translational Research Program, NUS Center for Nanomedicine, Yong Loo Lin School of Medicine, National University of Singapore, Singapore, 117597 Singapore

**Keywords:** Aggregation-induced emission (AIE), Assemblies, Radiosensitizer, Therapy, Fenton reaction

## Abstract

**Background:**

As cancer is one of the main leading causes of mortality, a series of monotherapies such as chemotherapy, gene therapy and radiotherapy have been developed to overcome this thorny problem. However, a single treatment approach could not achieve satisfactory effect in many experimental explorations.

**Results:**

In this study, we report the fabrication of cyclic RGD peptide (cRGD) modified Au_4_-iron oxide nanoparticle (Au_4_-IO NP-cRGD) based on aggregation-induced emission (AIE) as a multifunctional theranostic system. Besides Au_4_ cluster-based fluorescence imaging and enhanced radiotherapy, iron oxide (IO) nanocluster could realize magnetic resonance (MR) imaging and Fenton reaction-based chemotherapy. Abundant toxic reactive oxygen species generated from X-ray irradiation and in situ tumor-specific Fenton reaction under acidic microenvironment leads to the apoptotic and necrotic death of cancer cells. In vivo studies demonstrated good biocompatibility of Au_4_-IO NP-cRGD and a high tumor suppression rate of 81.1% in the synergistic therapy group.

**Conclusions:**

The successful dual-modal imaging and combined tumor therapy demonstrated AIE as a promising strategy for constructing multifunctional cancer theranostic platform.

**Graphical Abstract:**

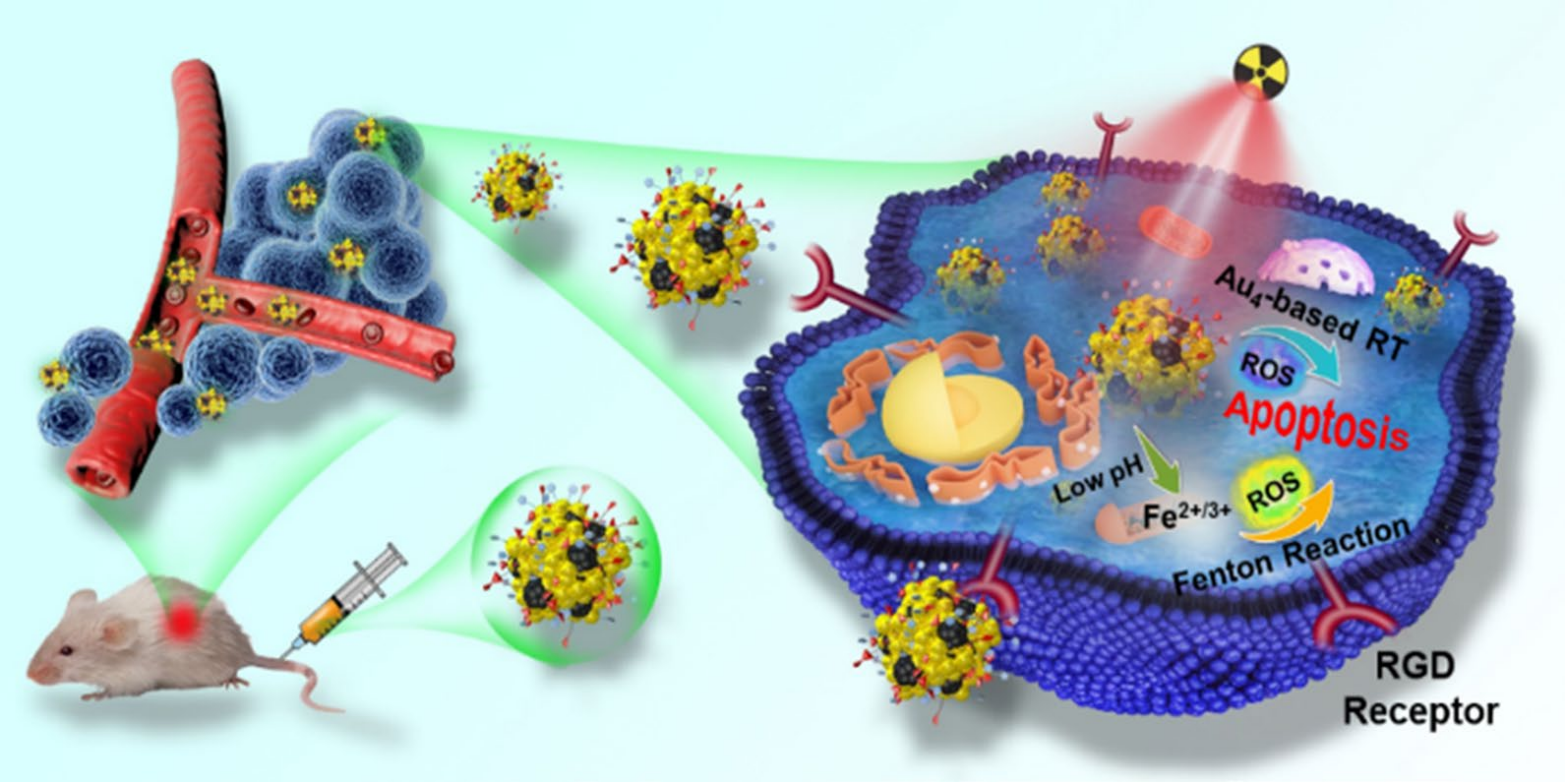

**Supplementary Information:**

The online version contains supplementary material available at 10.1186/s12951-021-01191-x.

## Introduction

Radiotherapy (RT) could precisely force high-energy X-ray radiation to destroy deep-seated orthotopic tumors. It has been widely used for treatment of local solid tumors such as breast, colorectal, esophageal, lung and brain tumors [1–3]. RT has been applied to over half the cancer patients globally and about 40% of cancer therapies involve the use of RT, sometimes in combination with other treatments [4–7]. A major and urgent challenge for efficient RT is how to maximize killing effect to cancer cells and in the meantime minimize damage to surrounding healthy tissues [8]. Among a wide range of agents that could affect the radiotherapy efficacy, gold-based nanomaterials remain a dominant candidate to be used for cancer treatment due to their high mass-attenuation coefficient and low cytotoxicity [9–12]. Besides classical gold nanoparticles (NPs) with large sizes (typically 10–50 nm), fluorescent gold nanoclusters (NCs) (1–3 nm) also emerged and displayed definite radiosensitizing effects [13–18]. For instance, Xie’s group explored glutathione (GSH) and bovine serum albumin (BSA)-protected Au_25_ NCs as radiosensitizers, demonstrating stronger enhancement for cancer radiotherapy than that of much larger Au NPs [19]. Most recently, Zang’s group reported a structurally defined gold-levonorgestrel nanocluster Au_8_(C_21_H_27_O_2_)_8_ as an effective radiosensitizer, pioneering the development of atomically precise Au NCs (with single crystal structure) based radiotherapy [20].

Reactive oxygen species (ROS) describes the chemical species formed upon incomplete reduction of oxygen, which mainly includes superoxide anion (O_2_^•−^), hydrogen peroxide (H_2_O_2_), singlet oxygen (^1^O_2_), and hydroxyl radical (•OH) [21]. ROS burst to disrupt the redox homeostasis in tumor cells is one of main mechanisms in cancer therapy [22–24]. It has been found that there is a relatively high concentration of H_2_O_2_ (∼100 μM) inside the tumor cells [25–28]. Therefore, a series of Fenton reaction-based therapeutic systems involving reductive metal ions and H_2_O_2_ to produce •OH have been developed [29–32]. For example, superparamagnetic iron oxide (IO) NPs could release iron ions (Fe^2+^/Fe^3+^) under acidic conditions and move into cytosol to further affect ROS homeostasis inside cells [33]. However, these cancer therapy strategies are often insufficient due to the limited H_2_O_2_ concentration and ion release rate. Accordingly, developing combination therapies based on several treatment methods is in great demand.

**Scheme 1:**
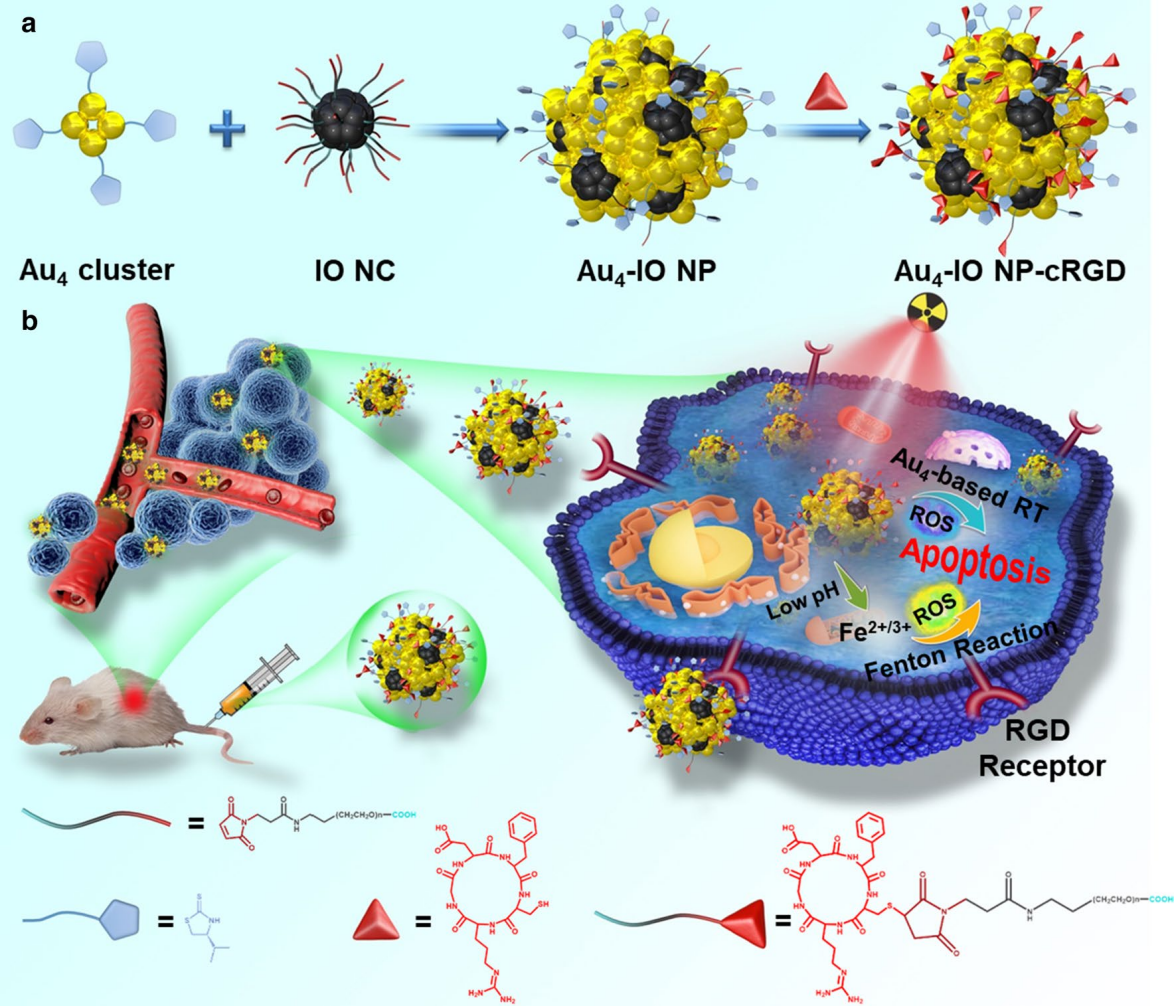
**a** Schematic illustration of the preparation of Au_4_-IO NP-cRGD. **b** Schematic illustration of the in vivo antitumor mechanism of Au_4_-IO NP-cRGD

As an efficient way to achieve bright emission from none-emissive single molecules, aggregation-induced emission (AIE) has embodied its superiority in bioimaging and cancer theranostics in the last decade [34–38]. Apart from AIE-active organic molecules, the specific AIE property has also been discovered in metal clusters in recent studies [39–43]. A representative example was chiral alkynyl ligands protected R/S-Cu_14_ NC, which was non-luminescent in dichloromethane (good solvent) but displayed red emission which was further enhanced with the increase of n-hexane (poor solvent) fraction [40]. The effectiveness of RT depends on the generation of ROS through radiolysis, and excessive ROS could overwhelm the antioxidant capacity of cells and cause cells death. In this case, we have studied the combination of RT and Fenton reaction to increase ROS yield through AIE technology. In addition, it has been demonstrated that AIEgens can also provide effective ROS generation in the aggregated state, which might promote energy transfer from the lowest excited singlet state (S1) to the lowest triplet state (T1) due to the prohibition of energy dissipation through non-radiative channels [44, 45]. Herein, we proposed green emissive Au_4_-IO NP assemblies based on AIE management in which non-fluorescent Au_4_ cluster solution assembled with IO NC in PBS buffer due to aurophilicity (Scheme 1a). With modification of cyclic arginine-glycine-aspartic-phenylalanine-cysteine acid (cRGD) peptide, Au_4_-IO NP-cRGD could achieve good targeting effect against α_v_β_3_-integrin overexpressing tumor cells. In the as-designed multifunctional Au_4_-IO NP-cRGD assemblies, Au_4_ cluster could produce abundant ROS upon X-ray irradiation, and IO NC could increase the amount of •OH based on Fenton reaction (Scheme 1b), finally resulting in synergistic irreversible damage to cancer cells. In the meantime, the fluorescence of aggregated Au_4_ cluster and magnetic property of IO NC could realize fluorescence and magnetic resonance (MR) imaging of tumors in vivo. The simple Au_4_-IO NP-cRGD assemblies based on AIE exhibited effective targeted Fenton reaction assisted enhanced radiotherapy.

## Experimental

### Synthesis of Au_4_-IO NP-cRGD

Au_4_ cluster (2 mM) was dissolved in DMF and then diluted with ethanol to 125 μM. 50 μM PEG modified-IO NC in PBS was added into ethanol solution of Au_4_ cluster with a volume ratio (IO NC: Au_4_ cluster) of 6:4 for uniform oscillation. For the conjugation of cRGD on the surface of Au_4_-IO NP, cRGD (10 mg/mL, dissolved in DMSO) was added into aqueous suspension of Au_4_-IO NP with uniform oscillation. Then, aqueous suspension was centrifuged (8000 rpm for 5 min) to remove free gold clusters and IO NC for further utilization.

### Cytotoxicity analysis

4T1 cells were seeded into 96-well plates at a density of approximately 10^4^ cells per well and cultured in RPMI-1640 medium supplemented with 10% fetal bovine serum at 37 °C in a humidified incubator with 5% CO_2_. After cell attachment, the cell medium was then replaced with 100 μL fresh medium containing different concentrations of Au_4_-IO NP/Au_4_-IO NP-cRGD (0, 1, 2.5, and 5 µM). The detailed dilution procedure: 2 mM Au_4_ cluster stock solution in DMF was firstly diluted with ethanol to 125 μM, and then 50 μM PEG modified-IO NC in cell culture medium was added into the above prepared Au_4_ cluster solution with a volume ratio (IO NC:Au_4_ cluster) of 6:4 to produce Au_4_-IO NP. After cRGD functionalization, Au_4_-IO NP-cRGD was diluted with cell culture medium for cytotoxicity assay. The control group was cell culture medium containing 0.04% DMF and 1.56% ethanol. After 24 h, CCK-8 reagent (10 μL per well) was added to the wells and incubated for 1 h. The absorbance at 450 nm was measured using a SpectraMax absorbance reader. The relative cell viability (%) was calculated as follows: (A_test_/A_control_) × 100.

### Cell imaging

4T1 cells were cultured on glass-bottomed Petri dishes at an initial density of 2 × 10^4^ cells/dish. 12 h later, the cell medium was replaced with fresh medium containing Au_4_-IO NP-cRGD or Au_4_-IO NP (Au_4_ content, 2 μM) for incubation. Confocal images of the cells were acquired with a Leica TCS SP8 confocal fluorescence microscope (excited at 405 nm).

### Colony formation assay

4T1 cells were seeded in cell culture dishes (35 mm × 12 mm) and incubated overnight for cell attachment. Then, the cells were divided into control group (without addition of nanomaterials) and treatment groups (2 μM Au_4_-IO NP/Au_4_-IO NP-cRGD). 6 h later, the cells were treated with different doses of X-ray (320 kV; 0, 2, 4, and 6 Gy) irradiation, and macroscopic cell colonies formed after 2 weeks. The cell colonies were fixed with 4% paraformaldehyde and stained with 0.2% crystal violet for counting. Each group was analyzed in triplicate and counted by Image J software. The cell survival fraction was calculated as the ratio of the number of colonies formed in treated wells to the number of colonies formed in untreated well (no materials and no X-ray). The cell survival curve was estimated by a multi-target single-hit model (S = 1 − (1 − e^−D/D0^)^n^ and then D_0_ and n were calculated by the Sigma Plot 12.0 software. The SER was expressed with the following formulae: SER = D_q_(control group)/D_q_(treated group) and D_q_ = ln(n) × D_0_, where D_q_ is the quasi-threshold dose. The control group was cell culture medium containing 0.04% DMF and 1.56% ethanol.

### Live/dead imaging

4T1 cells were cultured on glass-bottomed Petri dishes with an initial density of 4 × 10^4^ cells/dish. The same setup of cloning assay was applied with the live/dead assay until X-ray treatment. After X-ray irradiation, cells were cultured for another 24 h before viability assay. 4T1 cells were stained with a LIVE/DEAD cell imaging kit according to the manufacturer’s instructions. Confocal images were taken with a Leica TCS SP8 confocal fluorescence microscope (excited at 488 and 552 nm).

### Intracellular caspase-3/7 imaging

4T1 cells were seeded in glass-bottomed Petri dishes at an initial density of 2 × 10^4^ cells/dish overnight and incubated with nanomaterials Au_4_-IO NP/Au_4_-IO NP-cRGD of 2 μM for 6 h followed by irradiation at 0 and 4 Gy X-ray. After 24 h, the cells were stained with the CellEvent™ Caspase-3/7 Green Reagent. Confocal images were taken with a Leica TCS SP8 confocal fluorescence microscope (excitation: 488 nm).

### Flow cytometric assay

4T1 cells with a density of 2 × 10^5^ cells/well were first cultured in 6-well plates overnight. After complete attachment, the cells were incubated with Au_4_-IO NP/Au_4_-IO NP-cRGD (2 μM) for 6 h. Then, the cells were irradiated by X-ray (0 and 4 Gy) and further cultured for 24 h before washing and collection. Annexin V-FITC/PI double staining was used for apoptosis analysis using flow cytometry.

### Intracellular DNA break

4T1 cells were cultured on glass-bottomed petri dishes for incubation of materials and X-ray treatment. 24 h later, the cells were fixed with 4% paraformaldehyde and permeated with 0.25% Triton X-100. Then, the cells were blocked with 1% bovine serum albumin (BSA) for 1 h and incubated with γ-H2AX antibody (MA5-33062, Invitrogen) overnight at 4 °C. Ultimately, the secondary antibody Alexa Fluor™ 647 tag goat anti-rabbit IgG (H + L) (A21245, Invitrogen) was added for 1 ~ 2 h, and DAPI was used to stain cell nuclei. The cells were imaged using Leica TCS SP8 confocal fluorescence microscope at excitation wavelengths of 405 and 647 nm.

### Intracellular •OH detection

4T1 cells were cultured on glass-bottomed petri dishes and treated with or without Au_4_-IO NP/Au_4_-IO NP-cRGD (2 μM). After 6 h, the medium was removed, and then the prepared test solution (OH580 Stain Working solution) was added and incubated for 1 h at 37 °C. Then these groups were treated with or without X-ray (4 Gy). Finally, the cells were stained with DAPI, and imaged using Leica TCS SP8 confocal fluorescence microscope at excitation wavelengths of 405 and 540 nm.

### Animals and tumor model

All animal procedures were conducted in accordance with the Guide for Care and Use of Laboratory Animals and were approved by the Institutional Animal Care and Use Committee of the Zhengzhou University. BALB/c SPF mice (5-week-old females) were housed in a controlled environment with a 12 h/12 h light/dark cycle. A maximum of five animals were housed together and provided with food and water ad libitum. The mouse breast cancer cell line 4T1 is widely used as a syngeneic tumor model of breast cancer. 4T1 tumors were established by subcutaneously injecting approximately 5 × 10^6^ 4T1 cells. When the tumor reached a volume of ∼150 mm^3^, the mice were used for biodistribution and cancer therapy studies.

### Biodistribution

4T1 tumor-bearing mice were randomly divided into four groups followed by an intravenous injection of 100 μL of Au_4_-IO NP-cRGD (10 mg/kg). The mice were euthanized at 6, 24, 48 h, and 7 days after injection of materials, and the tumors and major organs were harvested. Then, the tumors and major organs were dispersed in 5 mL HNO_3_ overnight and digested with 5 mL of 30% H_2_O_2_ under boiling at 200 °C. The amounts of Au in each sample were measured using ICP-MS.

### In vivo imaging

Tumor-bearing mice were intravenous injected with Au_4_-IO NP-cRGD (10 mg/kg). Fluorescence imaging was conducted at 6 and 48 h post-injection using a PerkinElmer IVIS Spectrum in vivo imaging system. Imaging parameters were as follows: binning factor = 8, aperture = 2, exposure time = 2 s,excitation filter = 430, emission filter = 520. For in vivo MR imaging, tumor-bearing mice were anesthetized by isoflurane (3%) in oxygen and placed in an animal-specific body coil. MR images were acquired at 0, 6, 24, and 48 h after intravenous injection of Au_4_-IO NP-cRGD (10 mg/kg) or saline (as control) using a MR SOLUTIONS MRS-4717 system. Imaging parameters were as follows: echo spacing = 17, echo train = 7, slices = 18, slice thickness = 1 mm, FOV ratio = 1, FOV = 18 × 35 mm^2^.

### Cancer therapy

Murine breast cancer cell line 4T1 was widely used as a syngeneic tumor model of breast cancer, which was the tumor entity with the highest incidence in the world. 4T1 tumor-bearing mice were weighed and randomly divided into five groups: (1) Control, (2) Au_4_-IO NP-cRGD, (3) 4 Gy, (4) Au_4_-IO NP + 4 Gy, and (5) Au_4_-IO NP-cRGD + 4 Gy. Then, 100 μL of saline was intravenous injected into the mice in group 1 and 3, while the mice in group 2 and 5 or group 4 were intravenous injected with 100 μL Au_4_-IO NP-cRGD or Au_4_-IO NP (10 mg/kg) every 3 days for 14 days, respectively. Afterwards, the mice were subjected to X-ray irradiation 4 Gy at 48 h after injection. The mice were photographed and their body weights were measured every other day for 14 days. Also, the length (L) and width (W) of the tumors were measured to calculate the tumor volume (V) according to the following equation: V = L × W^2^/2.

### Evaluation of in vivo radiotherapy toxicity

At day 15 after the treatment, all mice were killed, and their peripheral blood and organs were collected for serum biochemistry analysis and pathological investigation respectively. The hearts, livers, spleens, lungs, kidneys, and tumors were fixed in 10% neutral formalin solution overnight. Then, the samples were embedded in paraffin blocks, sectioned into 5 μm slices, and mounted onto glass slides. After H&E staining, images were acquired with a Leica DMi1 inverted microscope.

### Statistical analysis

All data were collected in at least triplicate and represented as means ± SD. Differences between two groups were examined by the two-tailed Student's *t* test. Significant differences were noted with asterisks as *P* < 0.05 (*) or *P* < 0.01 (**).

## Results and discussion

### Preparation and characterization of Au_4_-IO NP-cRGD

**Fig. 1:**
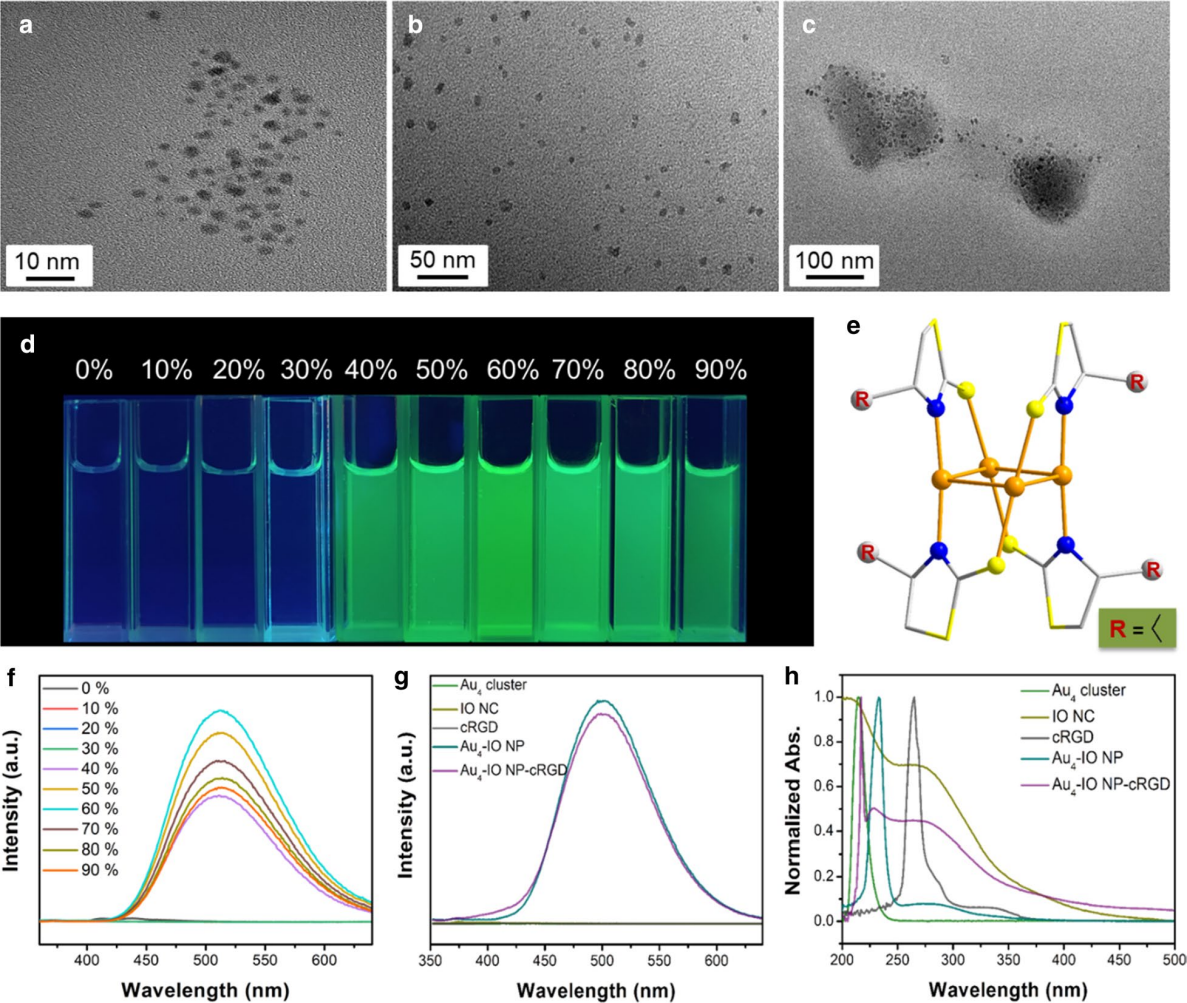
TEM images of **a** Au_4_ cluster, **b** PEG modified IO NC, and **c** Au_4_-IO NP. **d** Fluorescence photographs of Au_4_ cluster in dimethylformamide (DMF) with different fractions of IO NC in PBS under UV light. **e** The overall structures of Au_4_ cluster revealed by single-crystal X-ray analysis. Color codes: orange indicates Au, yellow indicates S, blue indicates N and gray indicates C. Hydrogen atoms were omitted for clarity. **f** Emission spectra of Au_4_ cluster in DMF with different fractions of IO NC in PBS. **g** Emission spectra and **h** UV–vis spectra of Au_4_ cluster, IO NC, cRGD, Au_4_-IO NP, and Au_4_-IO NP-cRGD

Au_4_(C_6_H_10_S_2_N)_4_ cluster and PEG modified iron oxide nanocluster (IO NC) were synthesized at a high yield according to published methods [46]. The phase purity of Au_4_ cluster was confirmed by powder X-ray diffraction (PXRD) (Additional file 1: Fig. S1). Coordination of the rhombic-like Au_4_ core was shown in Fig. 1e: Each ligand bridges one side of Au_4_ rhombus by a thiol S atom and an N atom from top and down. Intercluster Au–Au interactions and weak intercluster interactions among the ligands fasten the Au_4_ units, which collectively suppress nonradiative transitions and hinder intracluster motions in the aggregated state, resulting in aggregation-induced emission (AIE) [39]. Au_4_-IO nanoparticles (NPs) were acquired by mixing Au_4_ cluster in organic phase and IO NC in aqueous solution, then cRGD was linked to PEG ligand of IO NC for cancer cell targeting (Scheme 1a; details in the Experimental Section). The size of the Au_4_-IO NP-cRGD was approximately 100 nm, in which the sizes of Au_4_ cluster and IO NC were around 2 and 7 nm (Fig. 1a–c and Additional file 1: Fig. S2), respectively. Dynamic light scattering (DLS) measurements of the PEG modified IO NC and Au_4_ cluster (Additional file 1: Fig. S3a, b) also confirmed a relatively narrow size distribution with good dispersibility. AIE experiments showed that the non-emissive solution of Au_4_ cluster showed intense green emission centered at 510 nm when the fraction of poor solvent (H_2_O) rose to 40% (Fig. 1d, f). The fluorescence intensity continued to increase and plateaued at 60% H_2_O content. At the same time, the size increase of aggregates with increasing H_2_O fraction was also confirmed by DLS analysis (Additional file 1: Fig. S3). Notebly, in cell culture medium, the size of Au_4_-IO NP (IO NC: Au_4_ cluster = 6:4) is stable at about 100 nm, even if the assemblies were further diluted with cell medium (Additional file 1: Fig. S4). Au_4_-IO NP and Au_4_-IO NP-cRGD showed similar fluorescence spectra with emission peaks at approximately 510 nm (Fig. 1g). As shown in Fig. 1h, UV–vis spectrum of the as-fabricated Au_4_-IO NP-cRGD comprised all the characteristic absorbance peaks of Au_4_ cluster, IO NC, and cRGD (i.e., around 217 nm, 229 nm, and 263 nm). After conjugation of cRGD peptide, the zeta potential of NP increased from − 12.03 mV (Au_4_-IO NP) to 3.83 mV (Au_4_-IO NP-cRGD) (Additional file 1: Fig. S6). Moreover, ^1^H NMR and Diffusion Ordered NMR SpectroscopY (DOSY) also confirmed the successful modification of cRGD peptide (Additional file 1: Figs. S7, S8) [47]. At the same time, the stability of Au_4_-IO NP-cRGD in the blood was investigated, considering that they were intravenous injected (Additional file 1: Fig. S5). The fluorescence emission peak of Au_4_-IO NP-cRGD in serum exhibited little red-shift (510 nm to 520 nm). DLS measurements of Au_4_-IO NP-cRGD in serum confirmed a relatively narrow size distribution.

### Cytotoxicity and in vitro radiosensitizing effects

**Fig. 2:**
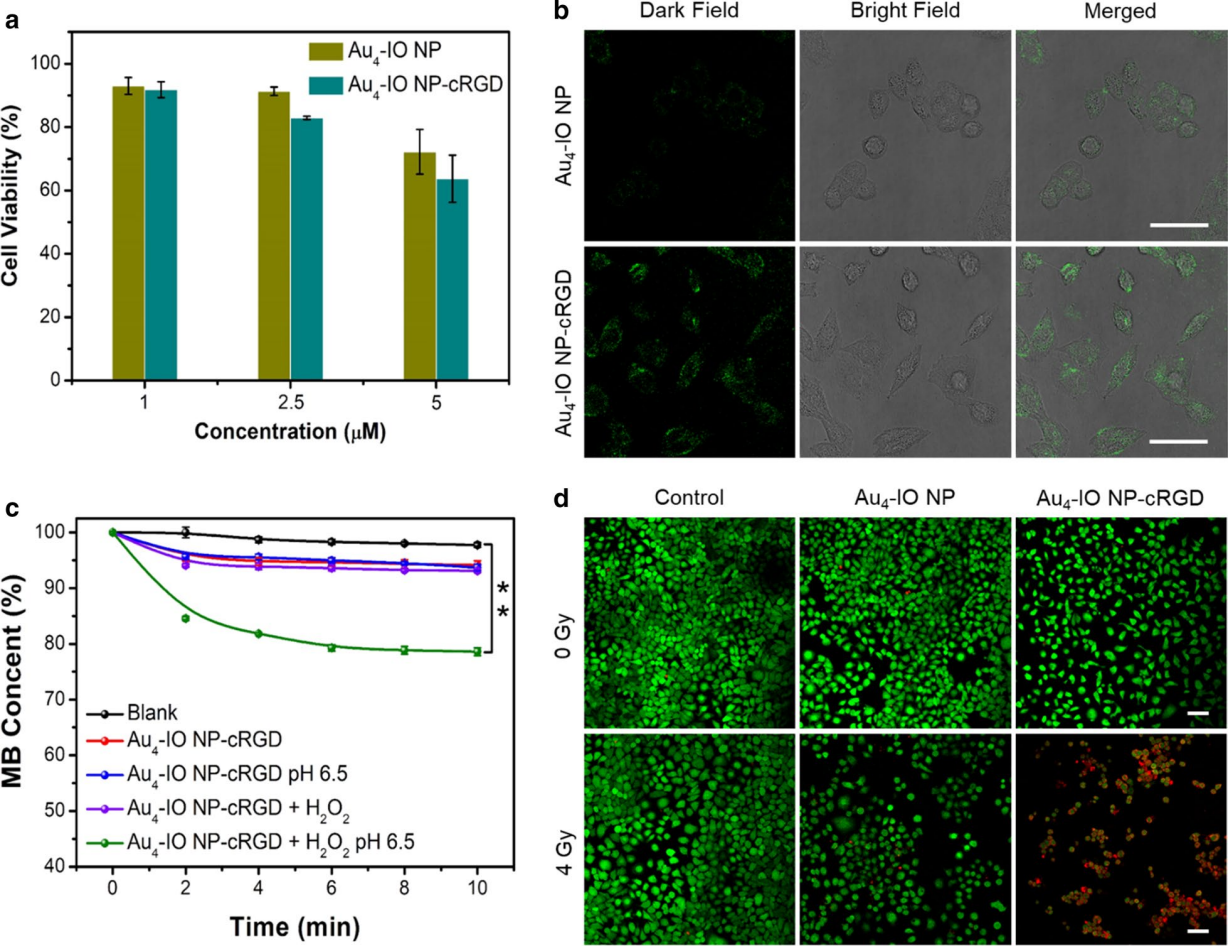
**a** In vitro cytotoxicity against 4T1 cells of Au_4_-IO NP/Au_4_-IO NP-cRGD. **b** Confocal imaging of 4T1 cells after incubation with Au_4_-IO NP and Au_4_-IO NP-cRGD (2 μM). Scale bar: 50 μm. **c** Time-dependent •OH generation from Au_4_-IO NP-cRGD with various treatments. **d** Live/dead imaging of 4T1 cells after receiving different treatments (control group, 4 Gy X-ray, Au_4_-IO NP, Au_4_-IO NP-cRGD, Au_4_-IO NP + 4 Gy X-ray, and Au_4_-IO NP-cRGD + 4 Gy X-ray). Green: live; red: dead. Scale bar: 50 μm. **P < 0.01

To evaluate the in vitro cytotoxicity of Au_4_-IO NP-cRGD as a radiosensitizer, we measured the relative cell viabilities of mouse breast cancer cells (4T1) with and without X-ray irradiation through a CCK-8 assay kit. As depicted in Fig. 2a, both the Au_4_-IO NP and Au_4_-IO NP-cRGD displayed a dose-dependent cytotoxicity against 4T1 cells, and Au_4_-IO NP-cRGD was slightly more toxic due to the targeting effect of cRGD. Additional X-ray irradiation caused enhanced proliferation inhibition of cells treated with Au_4_-IO NP/Au_4_-IO NP-cRGD (Additional file 1: Fig. S10). Confocal imaging results revealed that cells incubated with Au_4_-IO NP-cRGD (2 μM, IC_10_) showed brighter fluorescence than that of Au_4_-IO NP treated cells (Fig. 2b) and flow cytometry showed that the fluorescence intensity of Au_4_-IO NP-RGD treated cells was higher than that treated with Au_4_-IO NP (Additional file 1: Fig. S9), demonstrating more efficient cellular uptake owing to the effective molecular targeting capacity of cRGD to α_v_β_3_ integrin on the surface of cancer cells.[48–52]. In addition, there is no fluorescent signal in IO NC group. Due to the AIE property of Au_4_ cluster, a few fluorescent signals but weaker than Au_4_-IO NP-cRGD group could be seen in Au_4_ group (Additional file 1: Fig. S11). Besides gold based radiosensitization, •OH generated by Fenton reaction played a significant role in chemotherapy, initiated by free ferrous ions (Fe^2+^/Fe^3+^) from IO NC in this study. Methylene blue (MB) was employed to quantify the •OH production efficiency [25]. Compared with the Au_4_-IO NP-cRGD + H_2_O_2_ group (decreased to 93.1%) and Au_4_-IO NP-cRGD group at pH 6.5 (decreased to 93.7%), the UV absorbance of MB in the Au_4_-IO NP-cRGD + H_2_O_2_ group at pH 6.5 decreased to 78.6% (Fig. 2c), which was mainly due to the fact that Fe_3_O_4_ was decomposed under acidic conditions to produce Fe^2+^/Fe^3+^, and Fe^2+^ could react with H_2_O_2_ to produce toxic •OH. When cells were treated with 2 μM Au_4_-IO NP-cRGD along with the addition of 100 μM H_2_O_2_ in the acidified medium (pH 6.5), substantial cytotoxicity was observed due to Fenton reaction based •OH generation (Additional file 1: Fig. S12). Calcein acetoxymethylester (Calcine AM) and propidium iodide (PI) were used to monitor live (green) and dead (red) states of cells before and after X-ray irradiation [53]. As illustrated in Fig. 2d, cells of control and material only groups exhibited good viability, confirming the low toxicity of Au_4_-IO NP-cRGD at this concentration. Notably, death rate of cells incubated with Au_4_-IO NP-cRGD upon X-ray irradiation displayed a dose-dependent increase (Fig. 2d, Fig. S13).

**Fig. 3:**
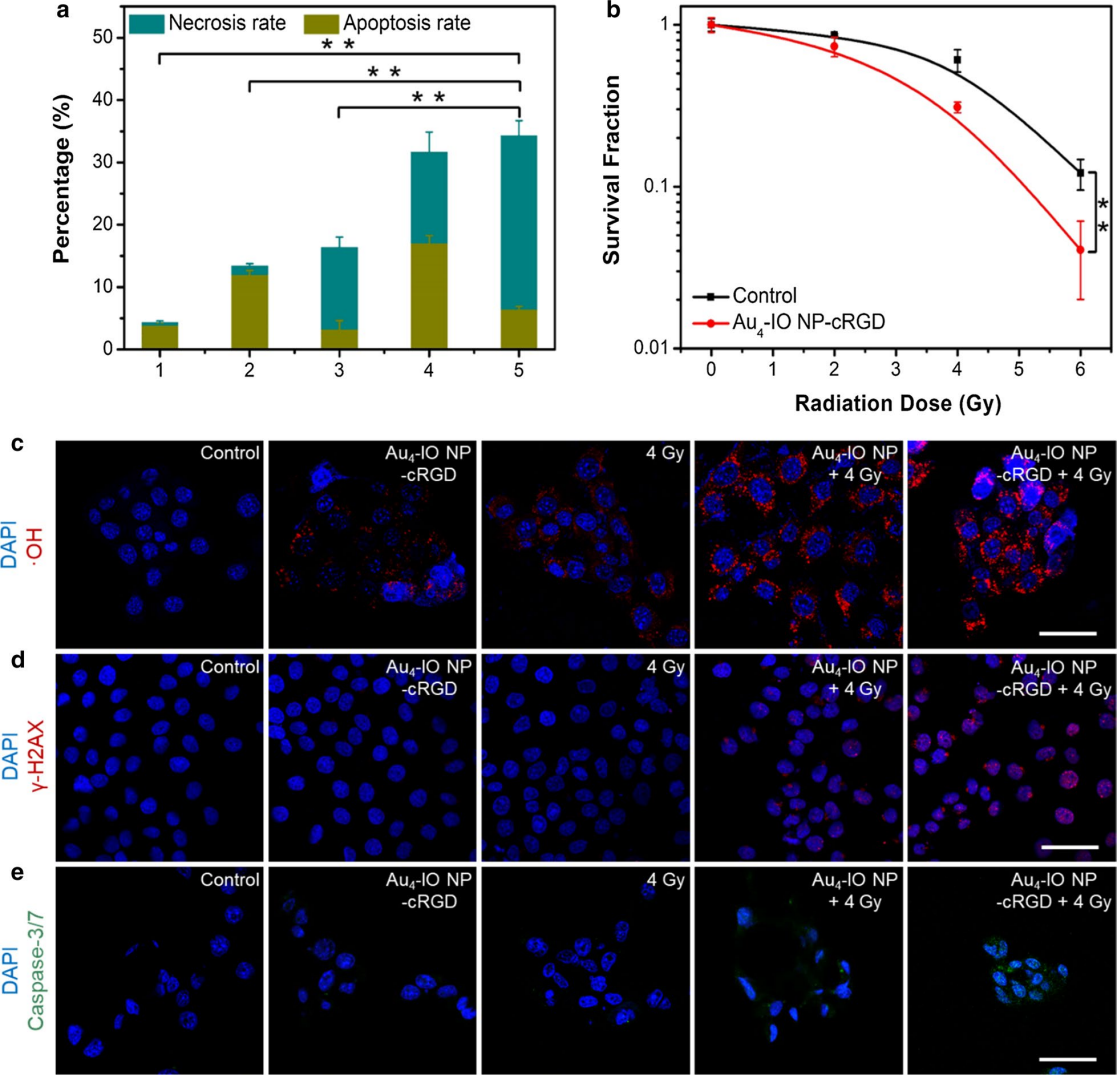
Statistical results of **a** the necrosis and apoptosis rate in different treatment groups: (1) Control, (2) Au_4_-IO NP-cRGD, (3) 4 Gy, (4) Au_4_-IO NP + 4 Gy, and (5) Au_4_-IO NP-cRGD + 4 Gy. **b** Survival curves of 4T1 cells received various treatments. Confocal fluorescence images of **c** •OH, **d** γ-H2AX, and **e** caspase 3/7 in 4T1 cells after different treatments (scale bar: 50 μm). **P < 0.01

Annexin V/PI assay [47] based on flow cytometry was performed to investigate cellular apoptosis during Au_4_-IO NP-cRGD enhanced RT. The sum of necrosis and apoptosis ratio of 4T1 cells treated by Au_4_-IO NP-cRGD or X-ray alone was 13.38% and 16.15%, respectively. (Fig. 3a and Additional file 1: Figs. S14–S17), while the combination of Au_4_-IO NP-cRGD and X-ray irradiation increased to 34.28%. Compared with Au_4_-IO NP + 4 Gy group, cells in Au_4_-IO NP-cRGD + 4 Gy group exhibited a higher necrosis rate. A cell colony formation assay was conducted to further assess the enhanced radiotherapy effect of Au_4_-IO NP-cRGD over a longer period of time. The corresponding results revealed similar dose-dependent radiosensitization effect (Fig. 3b, Additional file 1: Figs. S18, S19). The sensitization enhancement ratio (SER, calculated by SER = D_q_ (Control group)/D_q_ (Treated group)) [54, 55] of Au_4_-IO NP-cRGD for 4T1 cells was calculated to be 1.573 (Fig. 3b), which was slightly higher than that of the commonly used Au drug auranofin (1.462) [35]. In addition, the SER of Au_4_-IO NP for 4T1 cells was calculated to be 1.436 (Additional file 1: Fig. S19). These results suggest that the as-prepared Au_4_-IO NP-cRGD have a remarkable cell-killing ability under X-ray irradiation, making it a promising candidate for highly effective RT.

To further explore the therapeutic mechanism of Au_4_-IO NP-cRGD based RT, the cellular •OH generation after treatment with different NPs was first detected by a mitochondrial •OH detection assay kit [56]. As shown in Fig. 3c and Additional file 1: Fig. S20, the mean fluorescence intensity of •OH indicator in the Au_4_-IO NP-cRGD + X-ray treated group was slightly higher than that of Au_4_-IO NP + X-ray group, achieving 4.4 and 7.1 times of fluorescence intensity of 4 Gy and Au_4_-IO NP-cRGD group, respectively. Meanwhile, DNA damage of 4T1 cells after treating with Au_4_-IO NP-cRGD and X-ray was tested by immunofluorescence staining of γ-H2AX, a marker of double-stranded DNA damage [57, 58]. Very weak red fluorescence signals were detected in cells of control, Au_4_-IO NP-cRGD, and X-ray groups, representing the ignorable DNA break. Notably, 4T1 cells treated with Au_4_-IO NP-cRGD + X-ray exhibited the brightest red fluorescence signals, indicating a higher DNA damage level than Au_4_-IO NP + X-ray group (Fig. 3d, Additional file 1: Figs. S21, and S22). Afterwards, the activation of caspase-3/7 (apoptosis marker) was determined using Caspase-3/7 Green ReadyProbes™ Kit, which consisted of the DEVD peptide sequence conjugated to a nucleic acid-binding dye. In the presence of activated caspase 3/7, the dye would be cleaved from the DEVD peptide and became free to bind DNA, producing a fluorogenic response. Obvious fluorescence was detected in the Au_4_-IO NP-cRGD + 4 Gy X-ray group, and there was almost no green fluorescence in the control and Au_4_-IO NP-cRGD groups (Fig. 3e and Additional file 1: Fig. S23). Above mentioned results demonstrated that the as-prepared Au_4_-IO NP-cRGD could induce abundant •OH production, serious DNA damage and effective caspase 3/7 activation under X-ray irradiation, resulting in enhanced therapeutic efficiency of Au_4_-IO NP-cRGD based RT. Additionally, previouly reported Au NCs affected thioredoxin reductase (TrxR) activity could also be a possible mechanism for the synergetic treatment effect [35, 59].

## Biodistribution and in vivo therapy

**Fig. 4:**
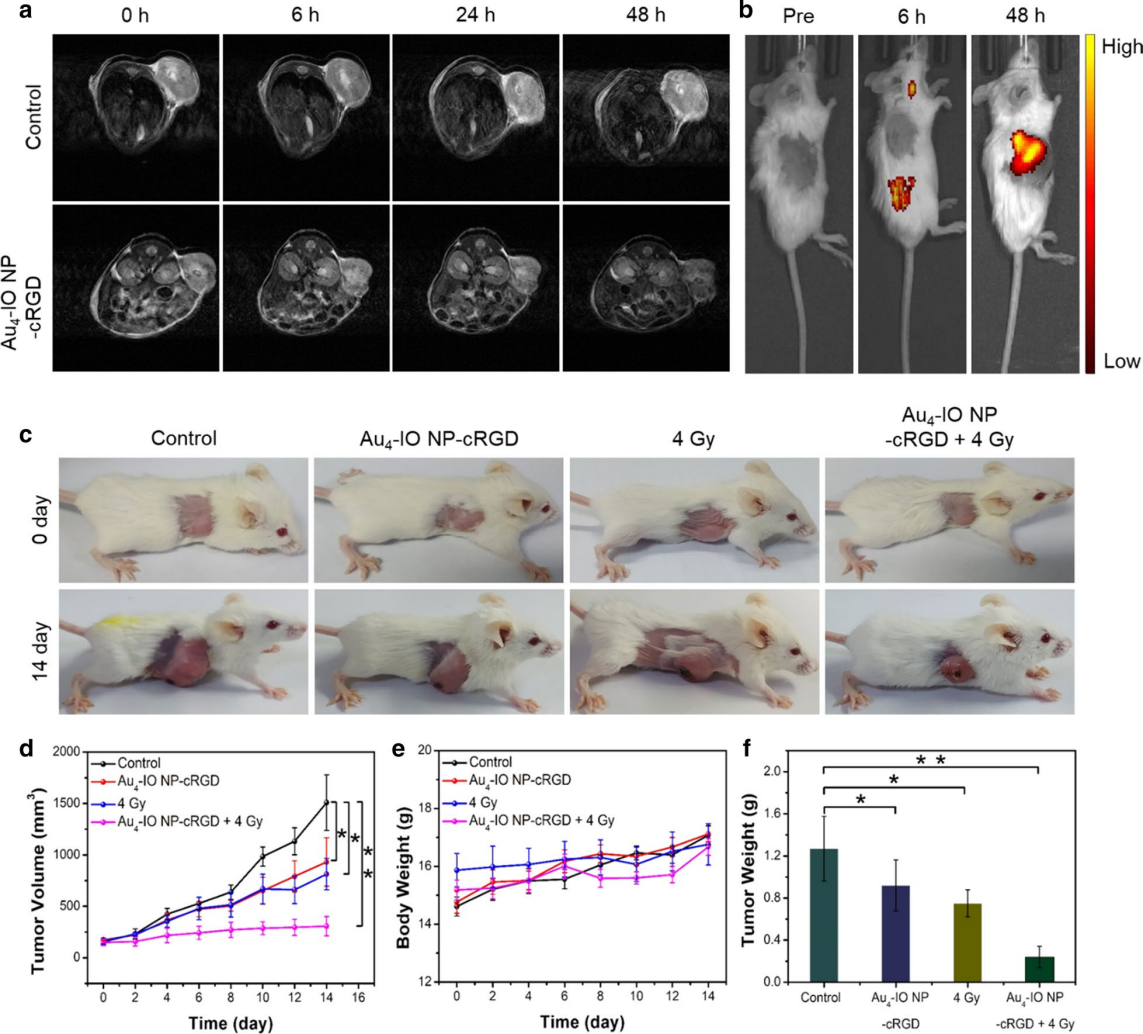
In vivo **a** MR images, and **b** fluorescence images acquired from tumor-bearing mice after intravenous injection of Au_4_-IO NP-cRGD at different time intervals. **c** Representative images of mice under various conditions at days 0 and 14. **d** Tumor volume curves of the mice. **e** Mouse body growth curve. **f** Statistical results of the tumor weights. *P < 0.05, **P < 0.01

Subsequently, in vivo performance of Au_4_-IO NP-cRGD in tumor imaging and therapy was explored. Firstly, the biodistribution of Au_4_-IO NP-cRGD in 4T1 tumor-bearing mice was monitored by inductively coupled plasma mass spectrometry (ICP-MS) via determination of Au. As shown in Additional file 1: Fig. S24, Au_4_-IO NP/Au_4_-IO NP-cRGD accumulation in the tumor site achieved maximum at 48 h post-injection (10 mg/kg). After intravenous injection of Au_4_-IO NP-cRGD or saline, MR imaging data were acquired at 0, 6, 24 and 48 h time points with a 4.7 T system. T2-weighted images displayed targeted signal from Au_4_-IO NP-cRGD in tumor (Fig. 4a). Then, we have tried fluorescence imaging due to the high QY of 40.5% in the aggregated state. Fluorescence imaging acquired by PerkinElmer IVIS system displayed similar result (Fig. 4b). Both MR and fluorescence imaging confirmed that Au_4_-IO NP-cRGD could effectively accumulate in 4T1 tumors. Following successful tumor-targeted imaging, we then investigated in vivo antitumor activities of Au_4_-IO NP-cRGD. Due to the weakly acidic extracellular tumor microenvironment and over expression of hydrogen peroxide in both intra- and extracellular tumor microenvironment, Au_4_-IO NP-cRGD could activate endogenous H_2_O_2_ to produce •OH in tumor site (Fenton reaction), playing a supplementary role in Au based enhanced RT. 4T1 tumor-bearing mice were treated with an experimental procedure as follows: mice were intravenous injected with Au_4_-IO NP-cRGD every three days and exposed to X-ray (4 Gy) at 48 h post injection (for 5 times in total). As shown in Fig. 4c, 4T1 tumors in control group (treated with saline) grew exponentially, while tumors treated with Au_4_-IO NP-cRGD and X-ray irradiation were significantly suppressed. Compared with the control group, the inhibition rates of tumor volume and tumor weight in synergistic therapy group reached 79.7% and 81.1%, respectively (Fig. 4d, f and Additional file 1: Fig. S25). In addition, the body weights of all mice were in normal range (Fig. 4e). In contrast, Au_4_-IO NP (lack of targeting function) resulted lower accumulation efficiency in tumors and relatively poor in vivo radiotherapy effect (Additional file 1: Fig. S26).

**Fig. 5:**
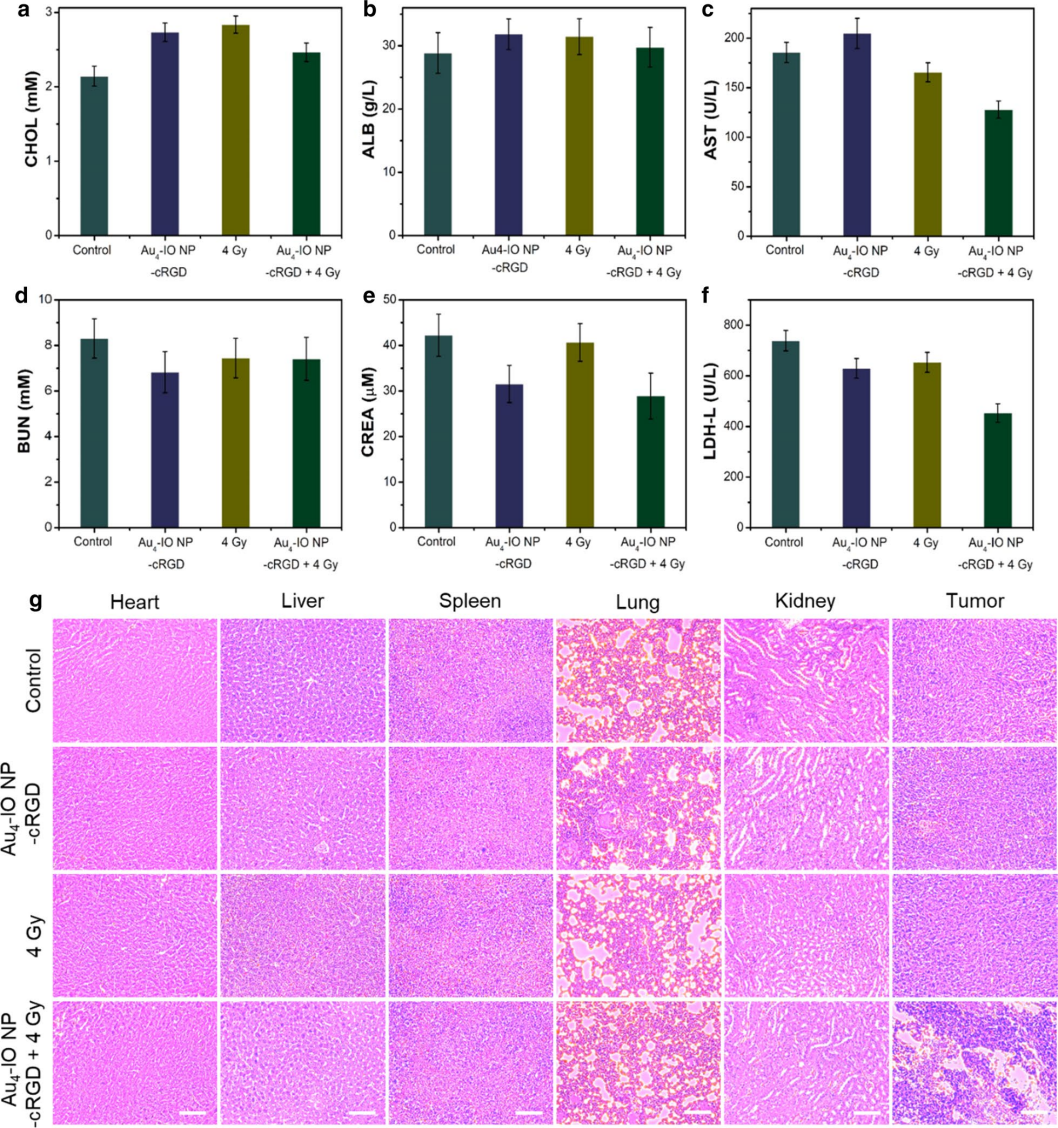
**a**–**f** Serum biochemistry analysis of tumor-bearing mice with different treatments: **a** cholesterol (CHOL), **b** albumin (ALB), **c** aminotransferase (AST), **d** blood urea nitrogen (BUN), **e** creatinine (CREA), and **f** lactate dehydrogenase (LDH-L). **g** H&E histological staining of excised organs and tumor slices. Scale bar: 200 μm

To assess the systemic toxicity of the as-prepared Au_4_-IO NP-cRGD in tumor treatment, serum biochemistry analysis and hematoxylin and eosin (H&E) staining of the main organ of mice were performed after 14 days treatment. Blood biochemical tests showed that the levels of cholesterol (CHOL), albumin (ALB), aminotransferase (AST), urea nitrogen (BUN), creatinine (CREA), and lactate dehydrogenase (LDH-L) in the Au_4_-IO NP-cRGD + X-ray group were similar to those in control group mice (Fig. 5a–f), and no histopathological abnormalities in the main organs were observed (Fig. 5g). In contrast, widespread damage was observed in the tumor tissue from the Au_4_-IO NP-cRGD + X-ray group compared with the other three groups. These data further demonstrated the significant synergistic therapy effect and in vivo biosafety of the as-prepared Au_4_-IO NP-cRGD in Fenton reaction-assisted enhanced radiation therapy.

## Conclusions

In summary, green emissive Au_4_-IO NP-cRGD with active cancer cell-targeting property was prepared based on AIE strategy to realize dual-modal imaging guided Fenton reaction-assisted enhanced radiotherapy. Firstly, the Au_4_ cluster composition in the assemblies played a vital role in enhancing the radiotherapy effect upon X-ray irradiation. Secondly, the IO NC composition in the assemblies could catalyze the in situ decomposition of the overexpressed H_2_O_2_ in tumor cells via Fenton reaction to produce •OH, further augmenting tumor therapy effect. ROS from these two parts resulted in DNA damage and up-regulation of caspase-3/7. In vivo therapy studies revealed a tumor suppression rate of 81.1% in the Fenton catalysis assisted enhanced radiotherapy. The successful dual-modal imaging and combined tumor therapy demonstrated AIE as a promising strategy for constructing multifunctional cancer theranostic platform.

## Supplementary Information

**Additional file 1: Fig. S1.** PXRD patterns of Au_4_ cluster. **Fig. S2.** TEM image of oil-phase IO NCs. **Fig. S3.** DLS spectra of (a) PEG modified IO NC and Au_4_ cluster in DMF with (b) 0% PBS, (c) 20% PBS, (d) 40% PBS, and (e) 60% PBS, (f) 80% PBS. **Fig. S4.** DLS spectra of (a) Au_4_-IO NP in DMF/ethanol with 60% medium and (b) 1000 times diluted Au_4_-IO NP using cell medium. **Fig. S5.** Emission spectra and (b) DLS spectra of Au_4_-IO NP-cRGD in serum at day 0, 3, 5, and 7. **Fig. S6.** Zeta potential of Au_4_-IO NP and Au_4_-IO NP-cRGD. **Fig. S7.** ^1^H-NMR spectra of cRGD-PEG-COOH. The characteristic peak of cRGD and PEG confirm the structure of cRGD-PEG-COOH. **Fig. S8.** ^1^H-NMR spectrum and DOSY spectrum of cRGD-PEG-COOH. **Fig. S9.** Flow cytometric assay of 4T1 cells after incubation with PBS, Au_4_-IO NP and Au_4_-IO NP-RGD. **Fig. S10.** In vitro cytotoxicity against 4T1 cells of Au_4_-IO NP and Au_4_-IO NP-cRGD with 4 Gy X-ray irradiation. **Fig. S11.** Confocal imaging of 4T1 cells after incubation with Au_4_ and IO NC. Scale bar: 50 μm. **Fig. S12.** Viability of 4T1 cells in different treatment groups: (1) Au_4_-IO NP-cRGD, (2) pH 6.5, (3) H_2_O_2_, (4) Au_4_-IO NP-cRGD pH 6.5, (5) Au_4_-IO NP-cRGD + H_2_O_2_, and (6) Au_4_-IO NP-cRGD + H_2_O_2_ pH 6.5. **Fig. S13.** Live/dead imaging of 4T1 cells after receiving different treatments (2 Gy X-ray, Au_4_-IO NP + 2 Gy X-ray, and Au_4_-IO NP-cRGD + 2 Gy X-ray). Green: live; red: dead. Scale bar: 50 μm. **Fig. S14.** Flow cytometric assay of 4T1 cells (negative control, positive control). **Fig. S15.** Flow cytometric assay of 4T1 cells with different treatments (batch 1). **Fig. S16.** Flow cytometric assay of 4T1 cells with different treatments (batch 2). **Fig. S17.** Flow cytometric assay of 4T1 cells with different treatments (batch 3). **Fig. S18.** Representative images of the colony formation assay of 4T1 cells with different treatments. **Fig. S19.** Survival curve of 4T1 cells received Au_4_-IO NP. **Fig. S20.** The fluorescence intensity of hydroxyl radical imaging of 4T1 cells (*λ*_*ex*_ = 540 nm) at 6 h after administering different treatments. (1) Control, (2) Au_4_-IO NP-cRGD, (3) 4 Gy, (4) Au_4_-IO NP + 4 Gy, and (5) Au_4_-IO NP-cRGD + 4 Gy. **Fig. S21.** Confocal images of γ-H2AX expression in 4T1 cells after receiving various treatments. Scale bar: 50 μm. **Fig. S22.** The fluorescence intensity of γ-H2AX imaging of 4T1 cells (*λ*_*ex*_ = 647 nm) at 6 h after administering different treatments. (1) Control, (2) Au_4_-IO NP-cRGD, (3) 4 Gy, (4) Au_4_-IO NP + 4 Gy, and (5) Au_4_-IO NP-cRGD + 4 Gy. **Fig. S23.** The fluorescence intensity of Caspase-3/7 imaging of 4T1 cells (*λ*_*ex*_ = 488 nm) at 6 h after administering different treatments. (1) Control, (2) Au_4_-IO NP-cRGD, (3) 4 Gy, (4) Au_4_-IO NP + 4 Gy, and (5) Au_4_-IO NP-cRGD + 4 Gy. **Fig. S24.** The biodistribution of (a) Au_4_-IO NP and (b) Au_4_-IO NP-cRGD at 6 h, 24 h, 48 h, 72 h, and 7 d after intravenous injection. **Fig. S25.** (a) Images of dissected tumors in control, Au_4_-IO NP-cRGD, and Au_4_-IO NP-cRGD + 4 Gy. (b) Images of dissected tumors in 4 Gy group. **Fig. S26.** (a) Representative images of mice of Au_4_-IO NP + 4 Gy group at days 0 and 14. (b) Tumor volume curve of the mice. (c) Mouse body growth curve. (d) Image of dissected tumors in Au_4_-IO + 4 Gy.

## Data Availability

All data analyzed during this study are included in this published article and its additional file.

## Abbreviations

cRGD: Cyclic RGD peptide
Au_4_-IO NP-cRGD: cRGD modified Au_4_-iron oxide nanoparticle
IO: Iron oxide
IO NC: Iron oxide nanocluster
AIE: Aggregation-induced emission
MR: Magnetic resonance
RT: Radiotherapy
NPs: Nanoparticles
NCs: Nanoclusters
GSH: Glutathione
BSA: Bovine serum albumin
ROS: Reactive oxygen species
DLS: Dynamic light scattering
DMF: Dimethylformamide
MB: Methylene blue
4T1: Mouse breast cancer cells
Calcine AM: Calcein acetoxymethylester
PI: Propidium iodide
H&E: Hematoxylin and eosin
CHOL: Cholesterol
ALB: Albumin
AST: Aminotransferase
BUN: Urea nitrogen
CREA: Creatinine
LDH-L: Lactate dehydrogenase
