# Supplementary material for: A multifunctional AIE gold cluster-based theranostic system: tumor-targeted imaging and Fenton reaction-assisted enhanced radiotherapy
Source: J Nanobiotechnology. 2021 Dec 20;19:438. doi: 10.1186/s12951-021-01191-x (PMC8686291; doi:10.1186/s12951-021-01191-x)
Supplement: Supplementary file 1 — Additional file 1: Fig. S1. PXRD patterns of Au4 cluster. Fig. S2. TEM image of oil-phase IO NCs. Fig. S3. DLS spectra of (a) PEG modified IO NC and Au4 cluster in DMF with (b) 0% PBS, (c) 20% PBS, (d) 40% PBS, and (e) 60% PBS, (f) 80% PBS. Fig. S4. DLS spectra of (a) Au4-IO NP in DMF/ethanol with 60% medium and (b) 1000 times diluted Au4-IO NP using cell medium. Fig. S5. Emission spectra and (b) DLS spectra of Au4-IO NP-cRGD in serum at day 0, 3, 5, and 7. Fig. S6. Zeta potential of Au4-IO NP and Au4-IO NP-cRGD. Fig. S7. 1H-NMR spectra of cRGD-PEG-COOH. The characteristic peak of cRGD and PEG confirm the structure of cRGD-PEG-COOH. Fig. S8. 1H-NMR spectrum and DOSY spectrum of cRGD-PEG-COOH. Fig. S9. Flow cytometric assay of 4T1 cells after incubation with PBS, Au4-IO NP and Au4-IO NP-RGD. Fig. S10. In vitro cytotoxicity against 4T1 cells of Au4-IO NP and Au4-IO NP-cRGD with 4 Gy X-ray irradiation. Fig. S11. Confocal imaging of 4T1 cells after incubation with Au4 and IO NC. Scale bar: 50 μm. Fig. S12. Viability of 4T1 cells in different treatment groups: (1) Au4-IO NP-cRGD, (2) pH 6.5, (3) H2O2, (4) Au4-IO NP-cRGD pH 6.5, (5) Au4-IO NP-cRGD + H2O2, and (6) Au4-IO NP-cRGD + H2O2 pH 6.5. Fig. S13. Live/dead imaging of 4T1 cells after receiving different treatments (2 Gy X-ray, Au4-IO NP + 2 Gy X-ray, and Au4-IO NP-cRGD + 2 Gy X-ray). Green: live; red: dead. Scale bar: 50 μm. Fig. S14. Flow cytometric assay of 4T1 cells (negative control, positive control). Fig. S15. Flow cytometric assay of 4T1 cells with different treatments (batch 1). Fig. S16. Flow cytometric assay of 4T1 cells with different treatments (batch 2). Fig. S17. Flow cytometric assay of 4T1 cells with different treatments (batch 3). Fig. S18. Representative images of the colony formation assay of 4T1 cells with different treatments. Fig. S19. Survival curve of 4T1 cells received Au4-IO NP. Fig. S20. The fluorescence intensity of hydroxyl radical imaging of 4T1 cells (λex = 540 nm) at 6 h after administ [file 12951_2021_1191_MOESM1_ESM.docx]

**Additional Information**

**A multifunctional** **AIE gold cluster-based theranostic system: tumor-targeted imaging and Fenton reaction-assisted enhanced radiotherapy**

Yue Hua^1^, Yuan Wang^1^, Xue Kang^1^, Fan Xu^1^, Zhen Han^1^, Chong Zhang^1^, Zhao-Yang Wang^1^, Jun-Qi Liu^2^, Xueli Zhao^1,^*, Xiaoyuan Chen^3,4,5,^*, Shuang-Quan Zang^1,^*

**Materials and methods**

**Materials**

Me_2_SAuCl was synthesized following a previously reported method[[1](#_ENREF_1)]. Iron (III) chloride hexahydrate (FeCl_3_·6H_2_O) was purchased from Sinopharm Chemical Reagent Co., Ltd. Sodium oleate was purchased from Energy Chemical Co., Ltd. Oleyl alcohol was purchased from Macklin^®^ Co., Ltd. Diphenyl ether and methylene blue trihydrate were purchased from Aladdin^®^ Co., Ltd. cRGD peptide and mPEG-Mal were purchased from Xi'an Ruixi Biological Technology Co., Ltd. Mouse breast cancer cells (4T1 cells) obtained from Cell Resource Center, Shanghai Institutes for Biological Sciences (Chinese Academy of Sciences). RPMI-1640 and fetal bovine serum (FBS) were purchased from Gibco™ Co., Ltd. DPBS (no calcium, no magnesium) was purchased from Mediatech, Inc. Trypsin-EDTA solution was purchased from Beijing Solarbio Science & Technology Co., Ltd. Cell culture flasks, cell culture plates and cell culture dishes were purchased from Nest Biotechnology Co., Ltd. 4',6-diamidino-2-phenylindole (DAPI) was purchased from Sigma-Aldrich. Triton^®^X-100 was purchased from Guangzhou Saiguo Biotech Co., Ltd. Cell Counting Kit-8 (CCK-8), Immunol Staining Fix Solution, and Crystal violet staining solution were purchased from Beyotime Biotechnology Co., Ltd. A LIVE/DEAD™ Cell Imaging Kit, Annexin V-FITC Apoptosis Detection Kit, CellROX™ Deep Red Reagent, CellEvent™ Caspase-3/7 Green ReadyProbes™ Kit, Highly Cross-Adsorbed Secondary Antibody Alexa Fluor™ 647 goat anti-rabbit IgG (H+L), and γ-H2AX antibody were purchased from Thermo Fisher Scientific. Mitochondrial Hydroxyl Radical Detection Assay Kit was purchased from Abcam Co., Ltd. BALB/c SPF mice (5-week-old females) were obtained from Beijing Vital River Laboratory Animal Technology Co., Ltd.

**Instrumentation**

Powder X-ray diffraction (PXRD) patterns of the samples were recorded on a D/MAX-3D diffractometer. TEM images were acquired with a Tecnai G2 F20 S-TWIN transmission electron microscope (operated at an acceleration voltage of 200 kV). 100/200 Duplex square hole copper mesh was used for TEM and the copper mesh was purchased from Beijing Zhongjing Science and Technology Co., Ltd. The sample preparation procedure as follows: first, the assembly was uniformly dispersed by ultrasonic. Then, we have used a pipette to pipette 10 μL of sample onto the copper net and dry it for later use. Dynamic light scattering (DLS) measurements were performed on a Horiba SZ-100 Nanoparticle Size Analyzer. UV-Vis spectroscopy was conducted using a TU-1901 double-beam UV-Vis spectrophotometer. Steady-state emission spectra of the compound were analyzed using a Horiba FluoroLog-3 spectrofluorometer. The absorbance of cells was analyzed using a SpectraMax^®^ Absorbance Reader. Confocal imaging was performed with a Leica TCS SP8 confocal fluorescence microscope. Cell flow cytometry was performed on a NovoCyte D3130 flow cytometer. ICP-MS analysis was performed with inductively coupled plasma mass spectrometer (ELEMENT 2). *In vivo* fluorescence imaging was analyzed on PerkinElmer IVIS Spectrum. The T2-weighted MR images were collected using a 4.7 T MR SOLUTIONS MRS-4717 (United Kingdom).

**Synthesis of iron-oleate complex**

The metal-oleate complex was prepared by reacting metal chlorides and sodium oleate. In a typical synthesis of iron-oleate complex, 5.4 g iron chloride (FeCl_3_·6H_2_O, 20 mmol) and 18.25 g sodium oleate (60 mmol) was dissolved in solvent composed of 40 mL ethanol, 30 mL distilled water and 70 mL hexane. The resulting solution was heated to 70 °C and kept at that temperature for four hours. When the reaction was completed, the upper organic layer containing iron-oleate complex was washed three times with distilled water in a separatory funnel. After washing, hexane was evaporated off, achieving iron-oleate complex in a waxy solid form.

**Synthesis of iron oxide nanoclusters (****IO NCs) in oil phase**

1.8 g (2 mmol) iron-oleate complex and 3.2 g oleyl alcohol (12 mmol) were dissolved in 10 g of diphenyl ether at room temperature. The reaction mixture was heated to 220 °C and then kept at that temperature for 30 min. When the reaction temperature reached 220 °C, a severe reaction occurred and the initial transparent solution became turbid and brownish black. The resulting solution containing iron oxide nanocrystals was then cooled to room temperature, and excess acetone was added to the solution to precipitate the nanocrystals. The nanocrystals were separated by centrifugation, producing IO NC in oil phase.

**Ligand exchange of IO NCs with maleimide-PEG**

The as-prepared IO NCs dispersed in tetrahydrofuran were mixed with tetrahydrofuran solution containing maleimide functionalized polyethylene glycol (PEG, MW=2000), followed by vigorous stir at 60 °C for 10-12 h, finally got the PEG-stabilized IO NC (IO NC).

**Preparation of (Au_4_L_4_)_n_ single crystals**

The ligand 4-isopropylthiazolidine-2-thione (16.1 mg, 0.1 mmol) and Me_2_SAuCl (29.5 mg, 0.1 mmol) were dissolved in 3 mL DCM and 1 mL CH_3_CN, then 10 μL triethylamine was added. The resultant solution was maintained at 4 °C for seven days to achieve colorless rod-like crystals. Yields: 64.1% for (Au_4_L_4_)_n_ based on Au.

**Detection of •OH**

•OH generated by Fenton reaction was detected by methylene blue (MB). Au_4_-IO NP-cRGD (50 μM) were added into the mixture containing 8 μg/mL MB and 100 μM H_2_O_2_. Absorbance of MB at 663 nm was measured by a SpectraMax absorbance reader to semi-quantitatively analyze the production of •OH at pH 6.5 and pH 7.4 respectively.


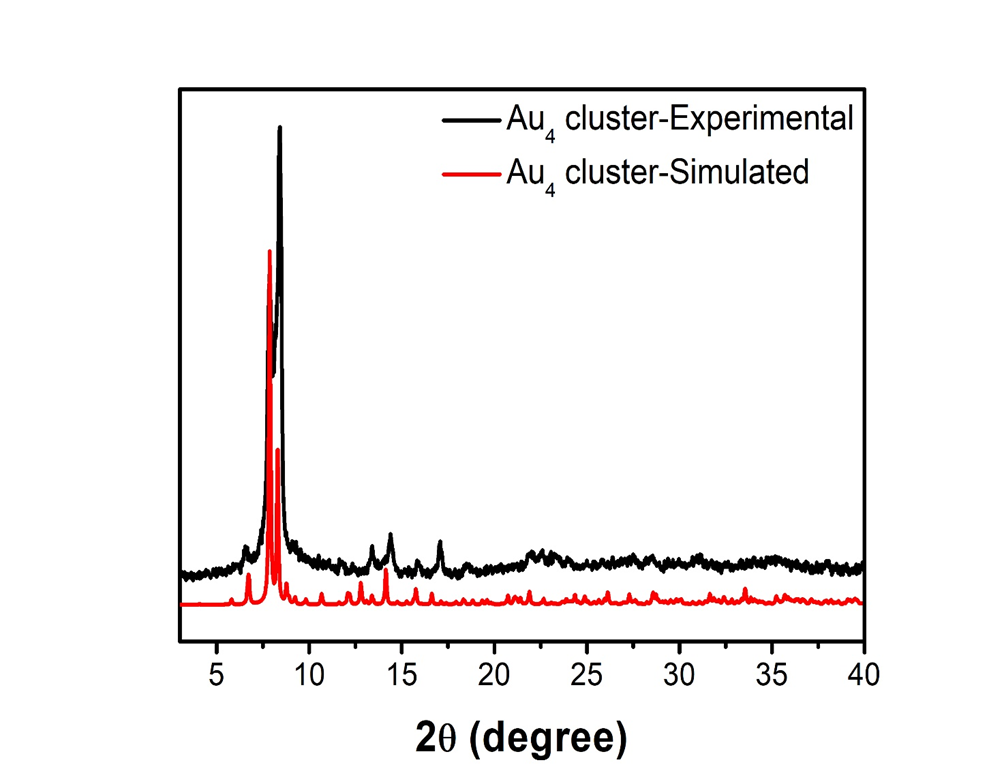


**Fig. S1** PXRD patterns of Au_4_ cluster.


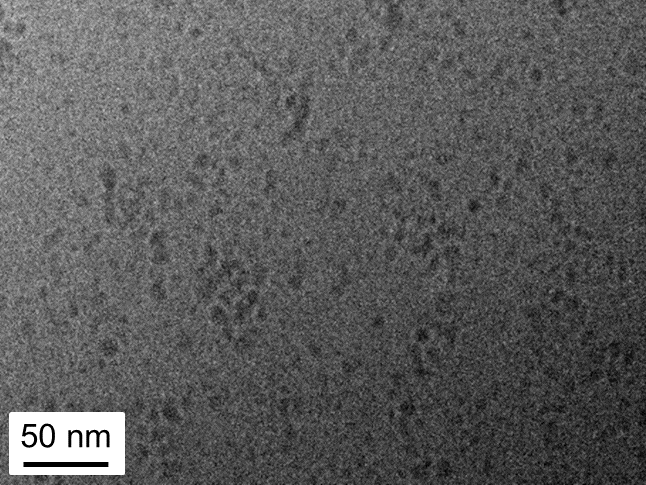


**Fig. S2** TEM image of oil-phase IO NCs.


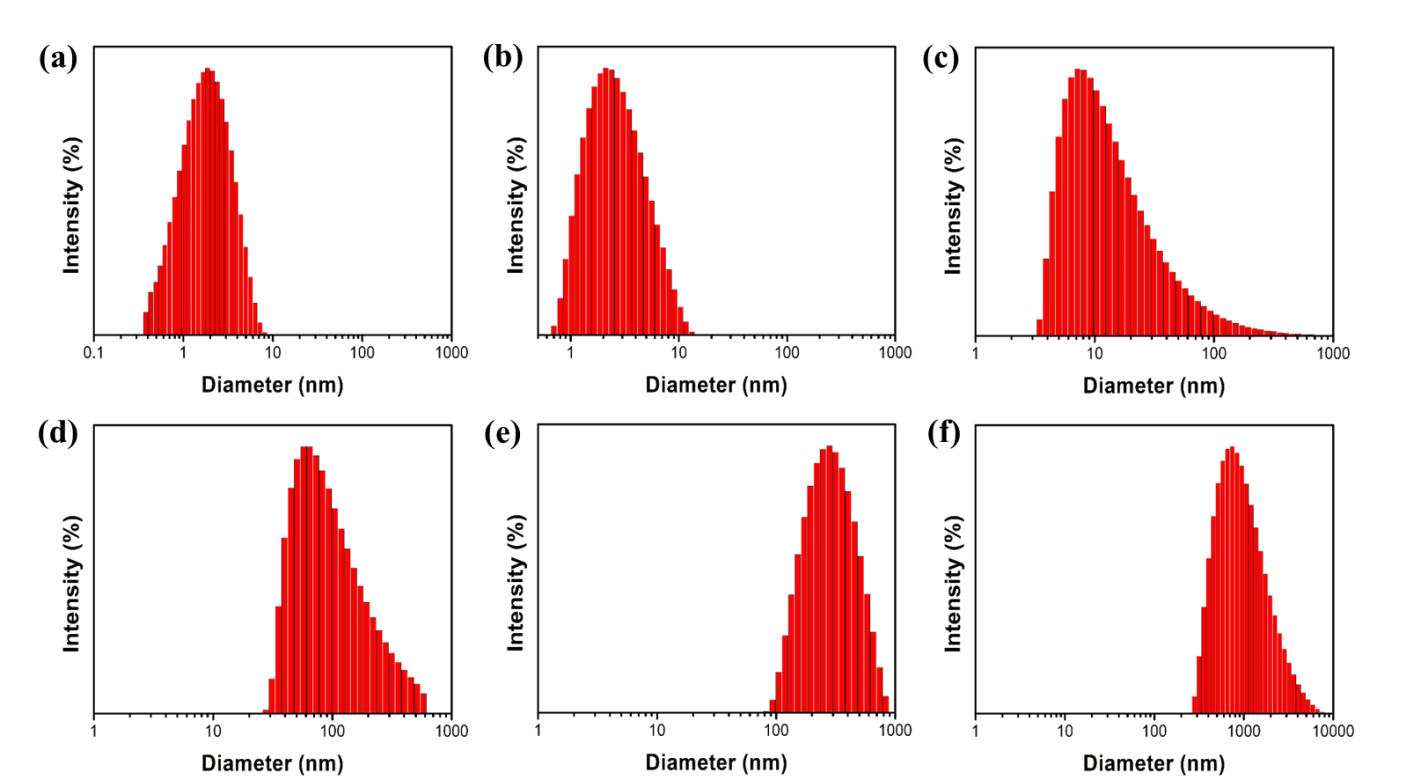


**Fig. S3** DLS spectra of (a) PEG modified IO NC and Au_4_ cluster in DMF with (b) 0% PBS, (c) 20% PBS, (d) 40% PBS, and (e) 60% PBS, (f) 80% PBS.


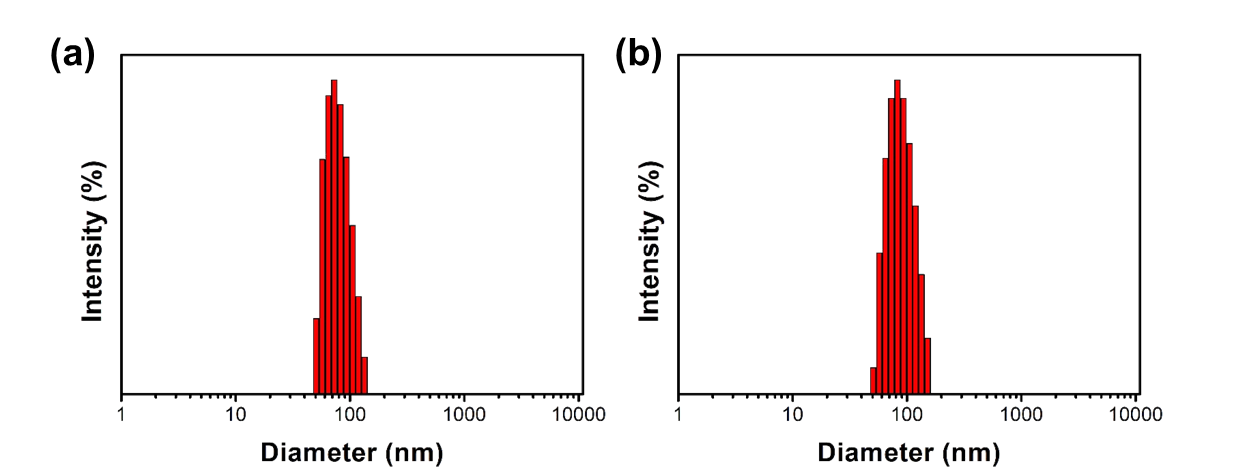


**Fig. S4** DLS spectra of (a) Au_4_-IO NP in DMF/ethanol with 60% medium and (b) 1000 times diluted Au_4_-IO NP using cell medium.


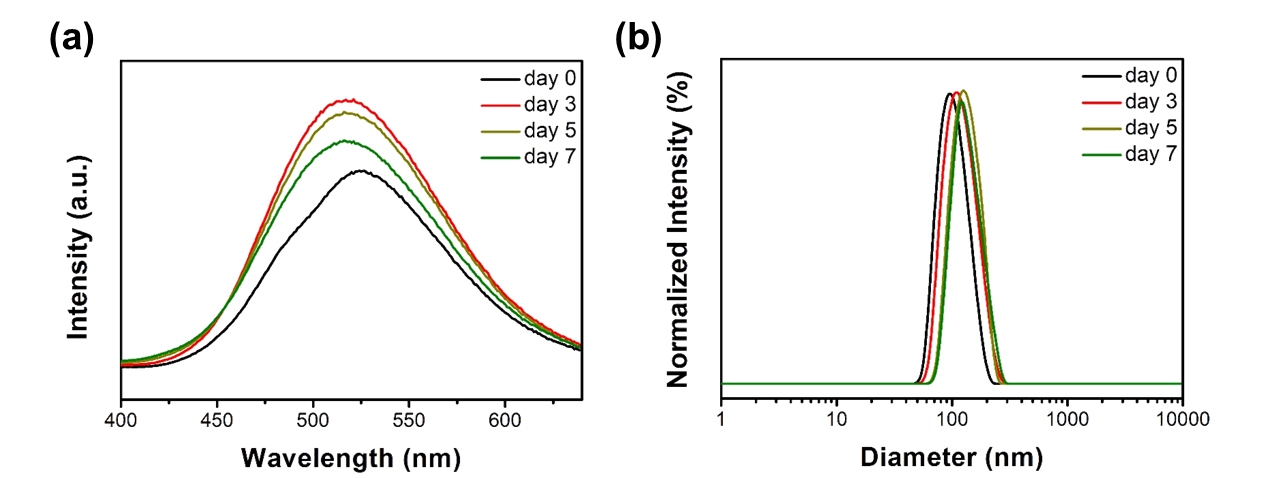


**Fig. S5** Emission spectra and (b) DLS spectra of Au_4_-IO NP-cRGD in serum at day 0, 3, 5, and 7.


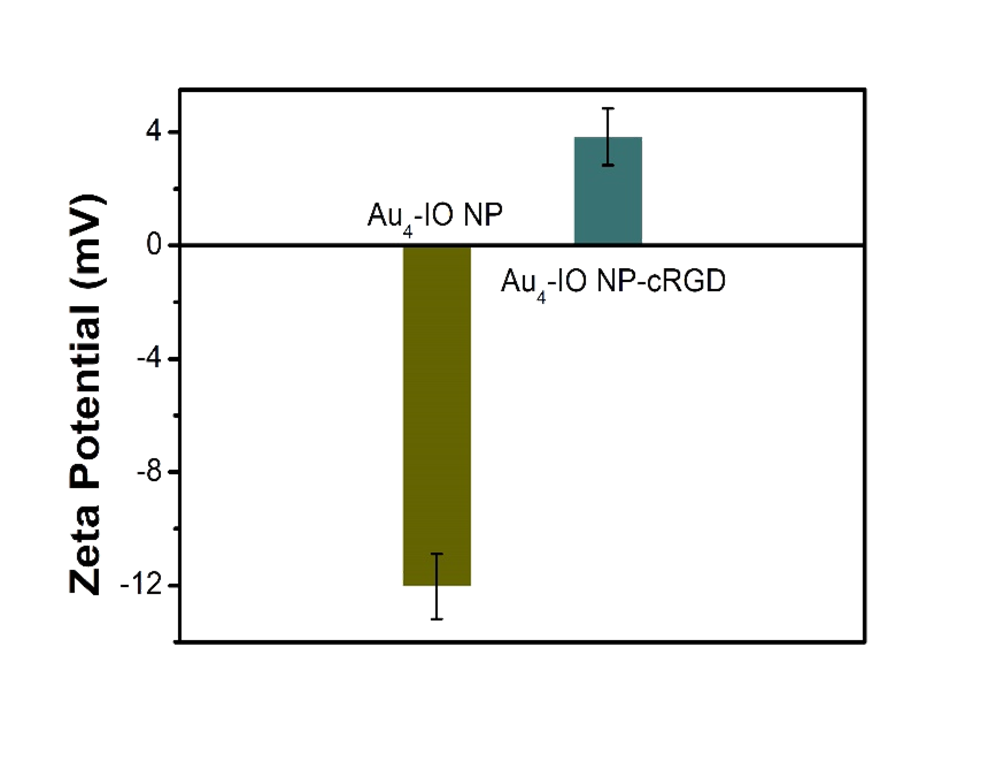


**Fig. S6** Zeta potential of Au_4_-IO NP and Au_4_-IO NP-cRGD.


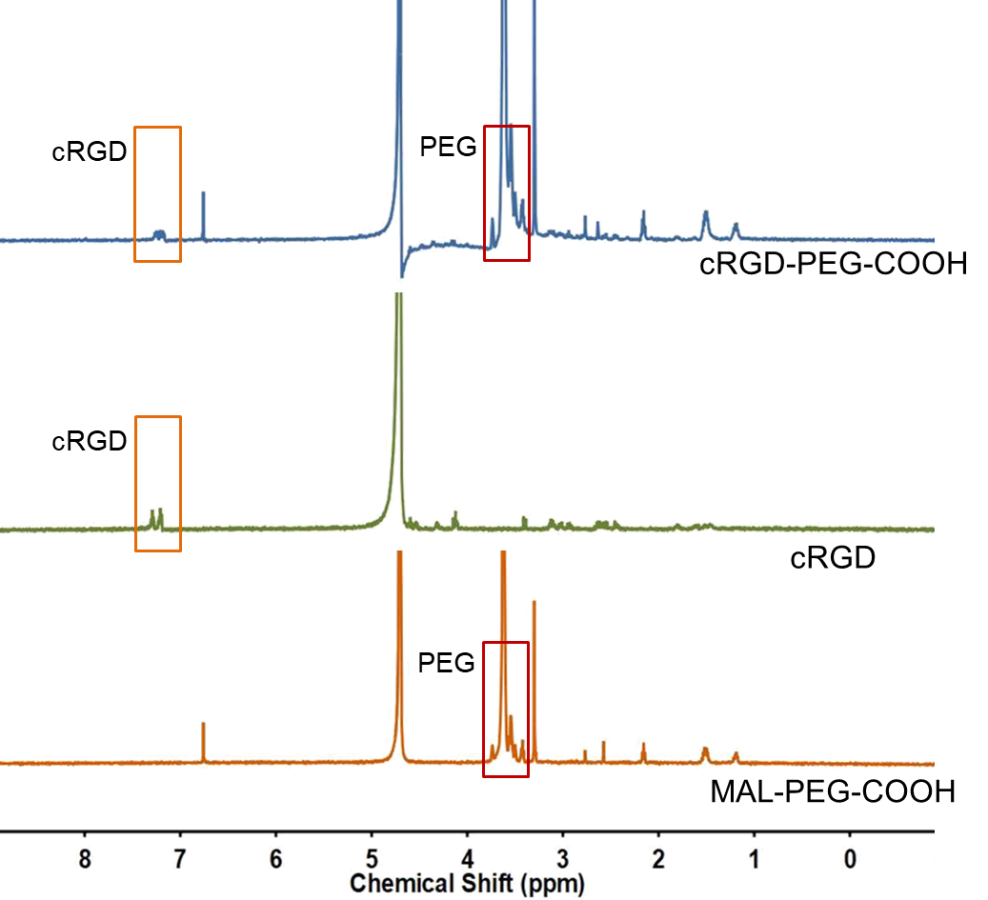


**Fig. S7** ^1^H-NMR spectra of cRGD-PEG-COOH. The characteristic peak of cRGD and PEG confirm the structure of cRGD-PEG-COOH.


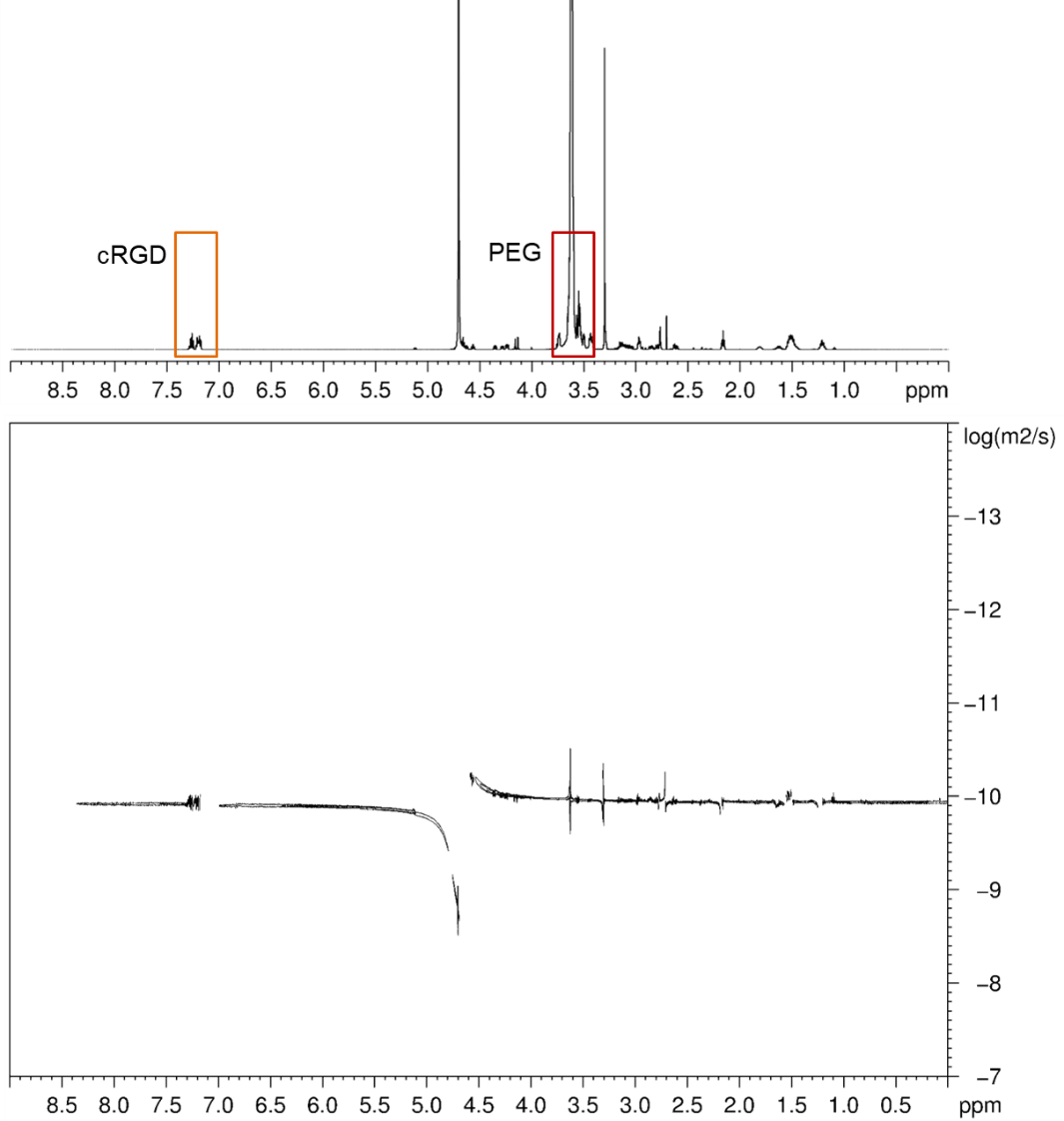


**Fig. S8** ^1^H-NMR spectrum and DOSY spectrum of cRGD-PEG-COOH.


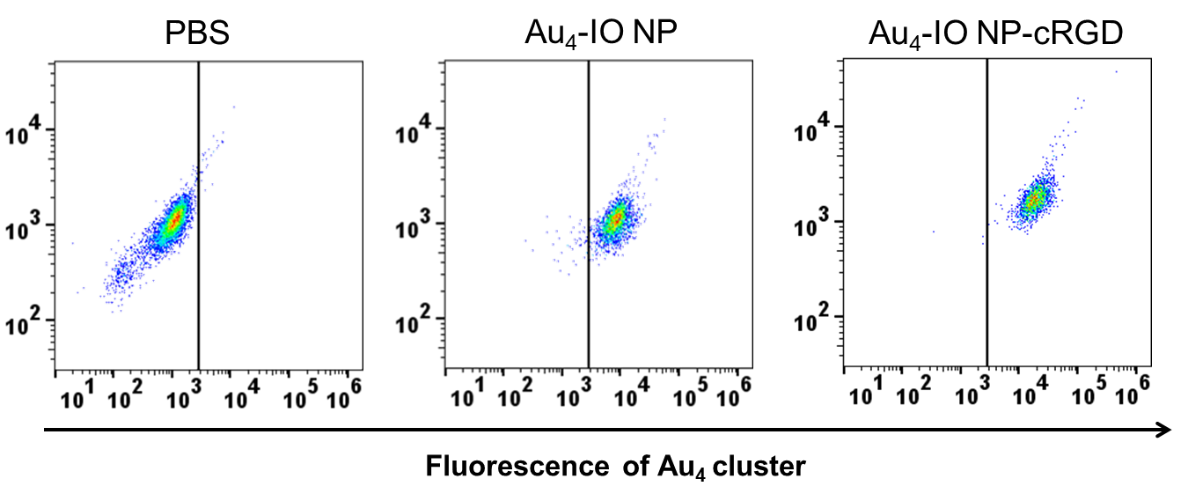


**Fig. S9** Flow cytometric assay of 4T1 cells after incubation with Au_4_-IO NP or Au_4_-IO NP-RGD.


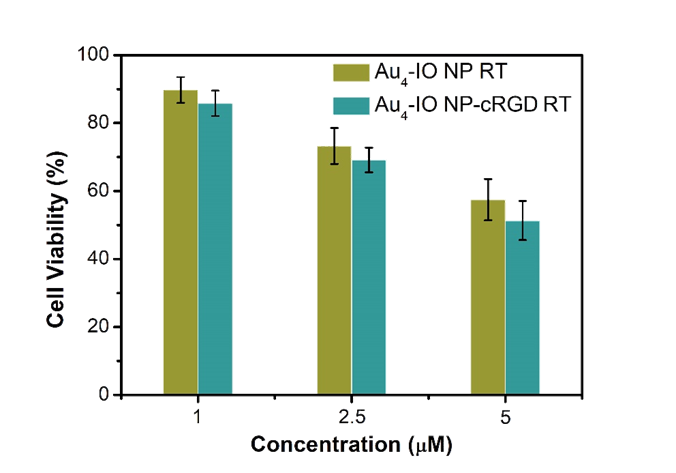


**Fig. S10** *In vitro* cytotoxicity against 4T1 cells of Au_4_-IO NP and Au_4_-IO NP-cRGD with 4 Gy X-ray irradiation.


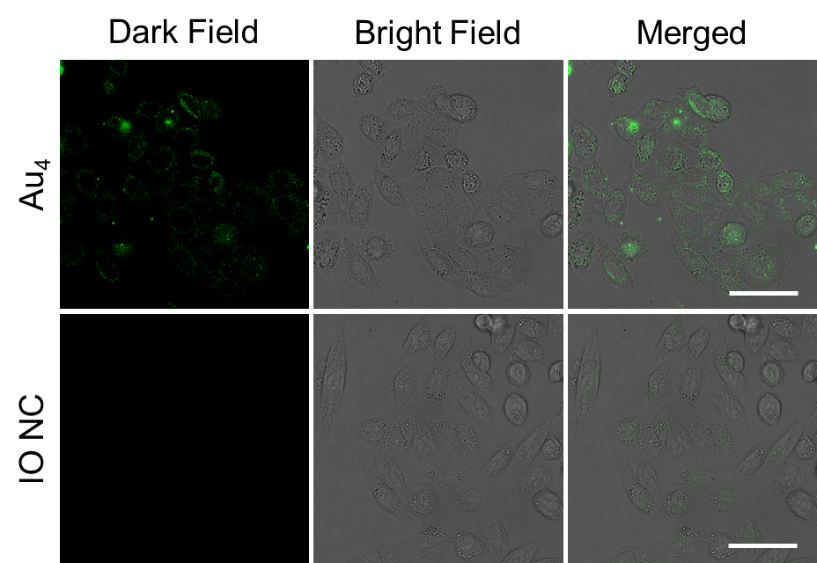


**Fig. S11** Confocal imaging of 4T1 cells after incubation with Au_4_ and IO NC. Scale bar: 50 μm.


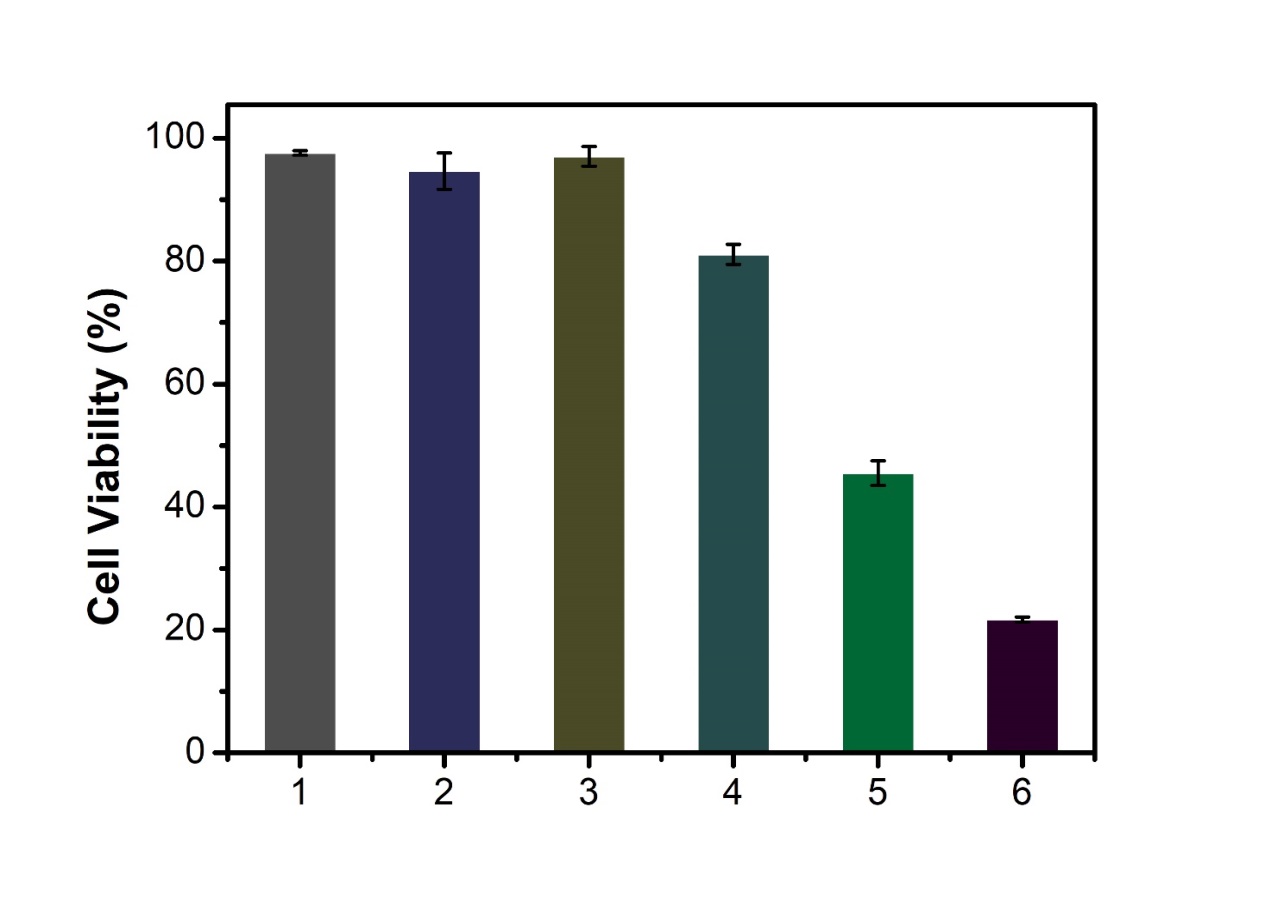


**Fig. S12** Viability of 4T1 cells in different treatment groups: (1) Au_4_-IO NP-cRGD, (2) pH 6.5, (3) H_2_O_2_, (4) Au_4_-IO NP-cRGD pH 6.5, (5) Au_4_-IO NP-cRGD + H_2_O_2_, and (6) Au_4_-IO NP-cRGD + H_2_O_2_ pH 6.5.


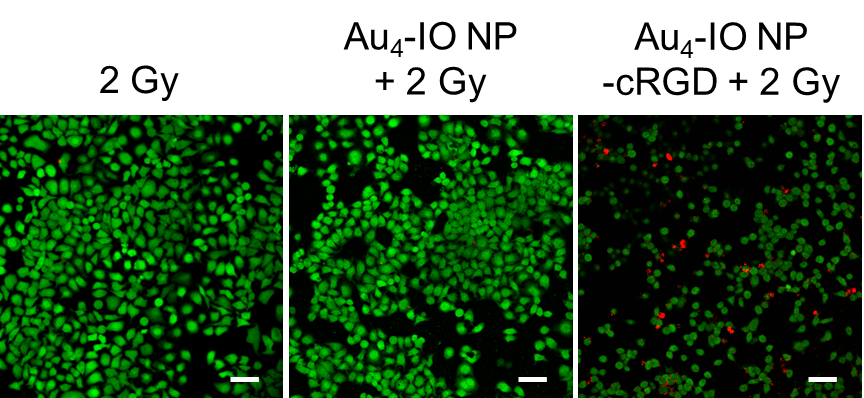


**Fig. S13** Live/dead imaging of 4T1 cells after receiving different treatments (2 Gy X-ray, Au_4_-IO NP + 2 Gy X-ray, and Au_4_-IO NP-cRGD + 2 Gy X-ray). Green: live; red: dead. Scale bar: 50 μm.


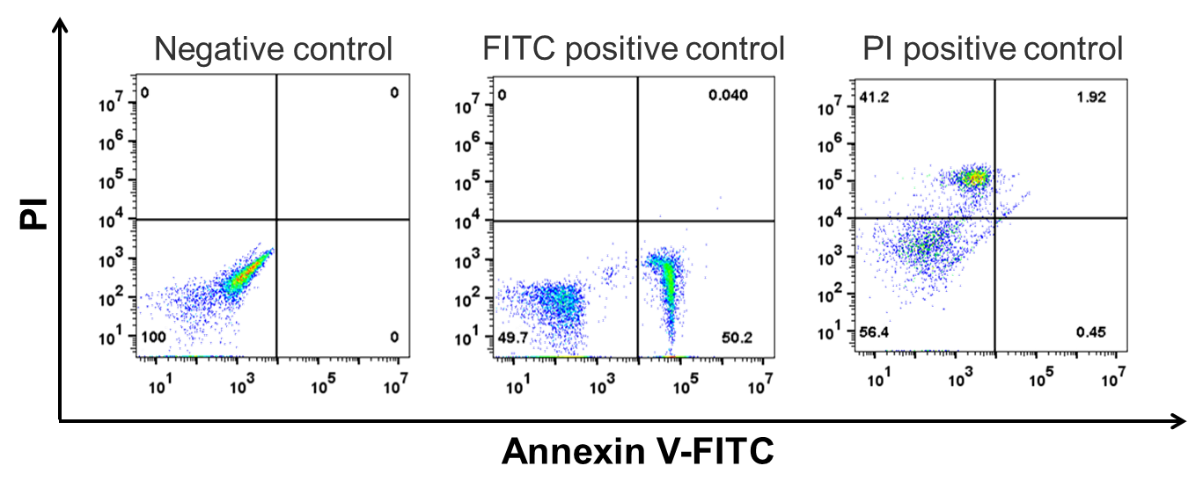


**Fig. S14** Flow cytometric assay of 4T1 cells (negative control, positive control).


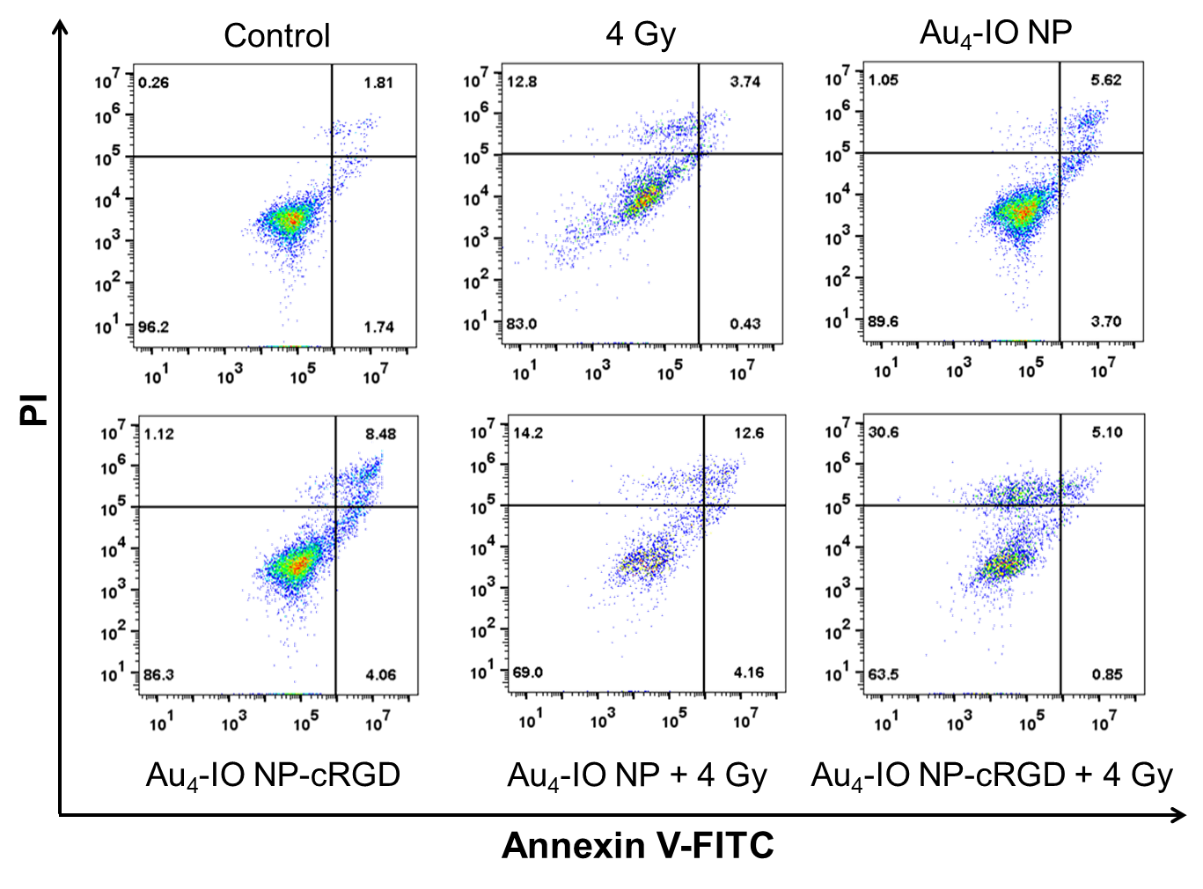


**Fig. S15** Flow cytometric assay of 4T1 cells with different treatments (batch 1).


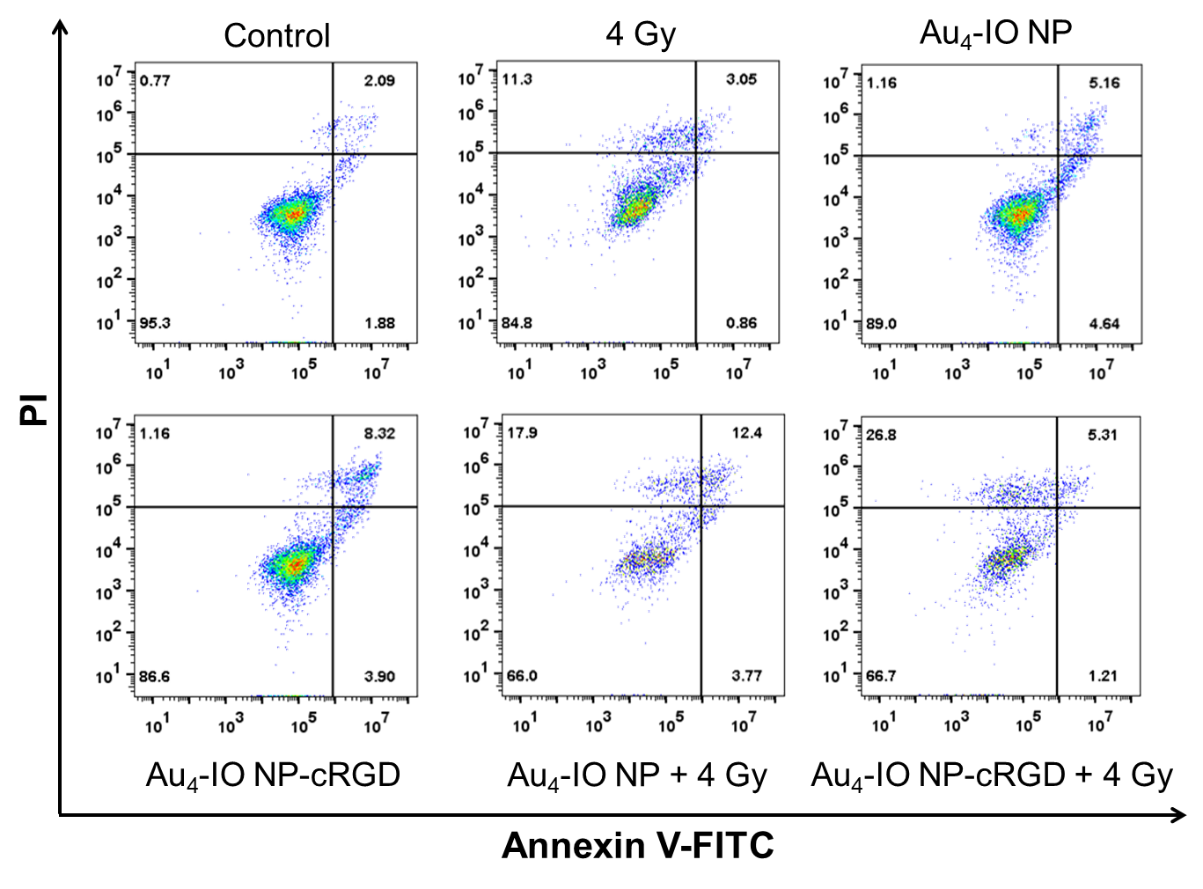


**Fig. S16** Flow cytometric assay of 4T1 cells with different treatments (batch 2).


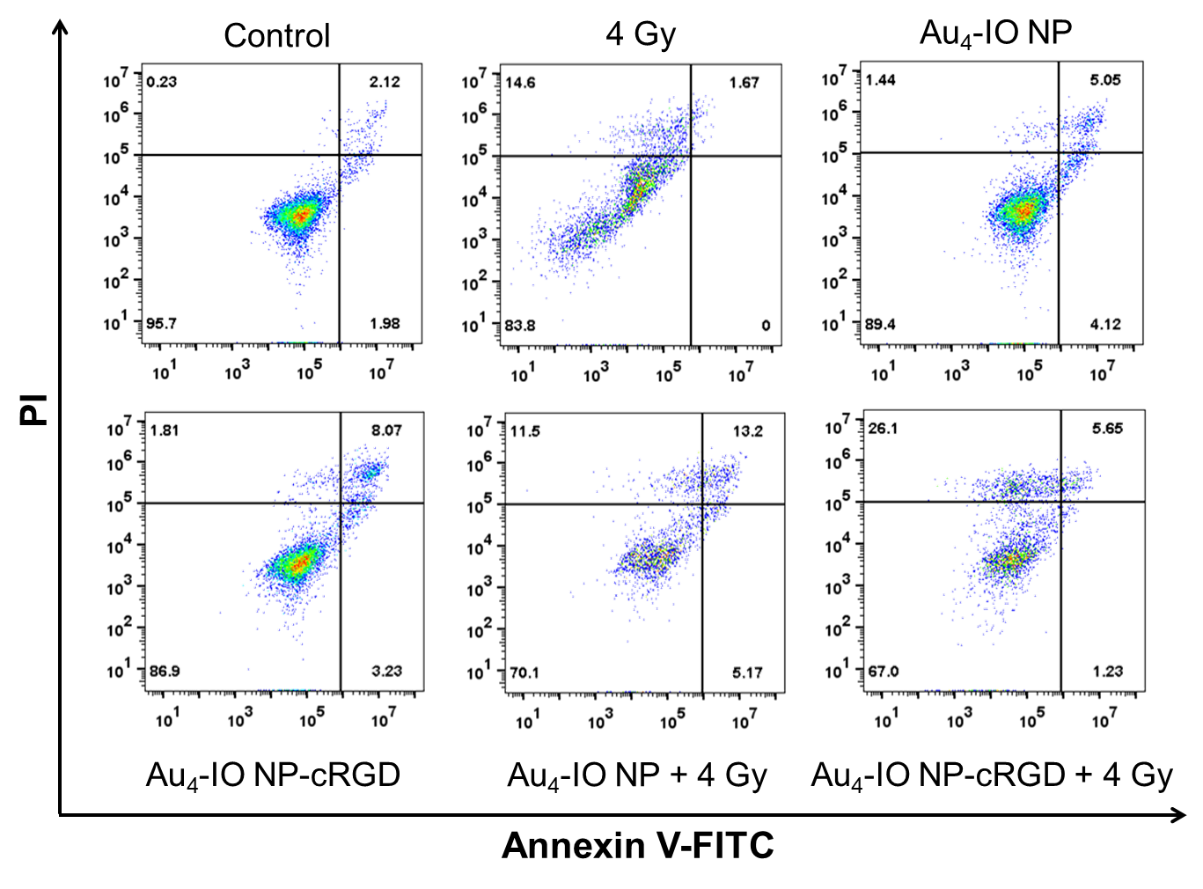


**Fig. S17** Flow cytometric assay of 4T1 cells with different treatments (batch 3).


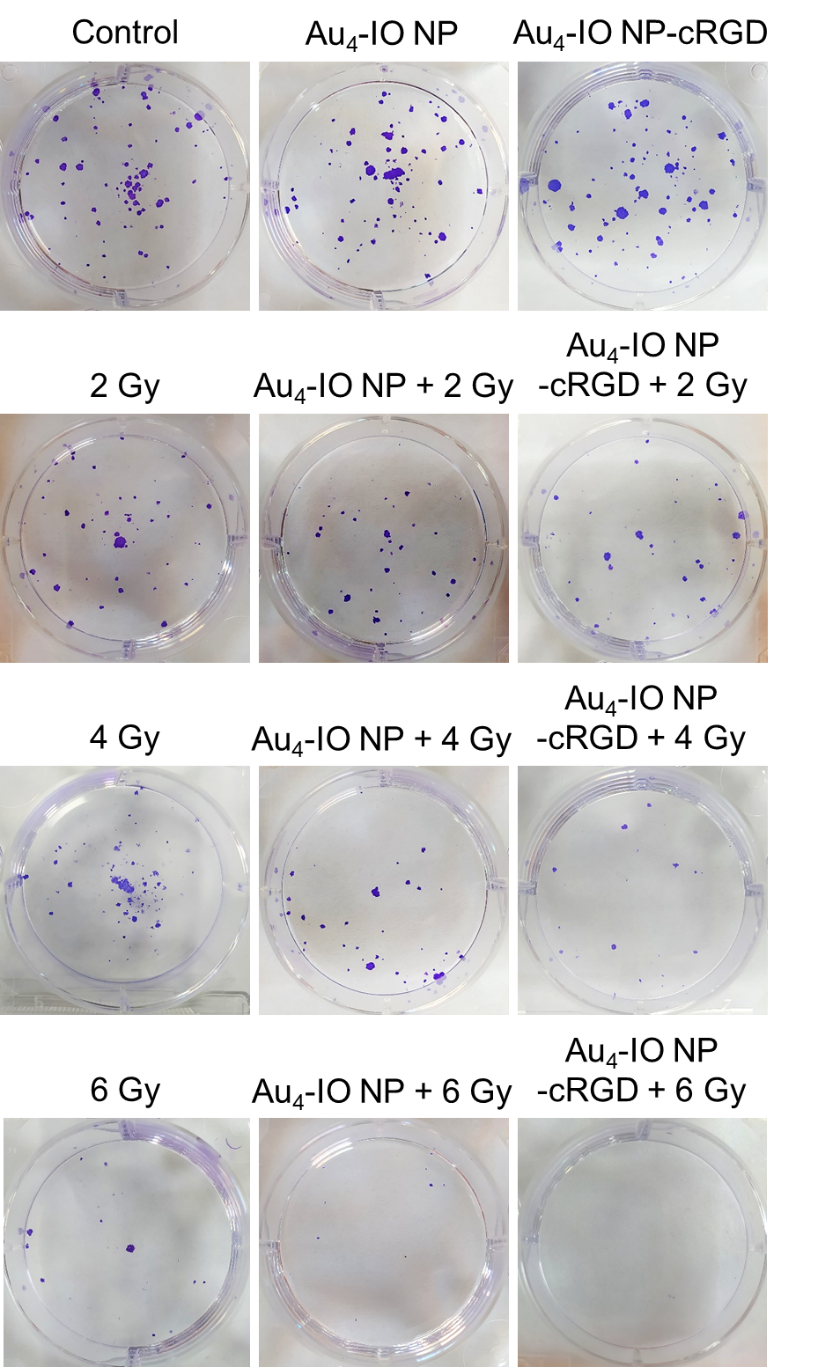


**Fig. S18** Representative images of the colony formation assay of 4T1 cells with different treatments.


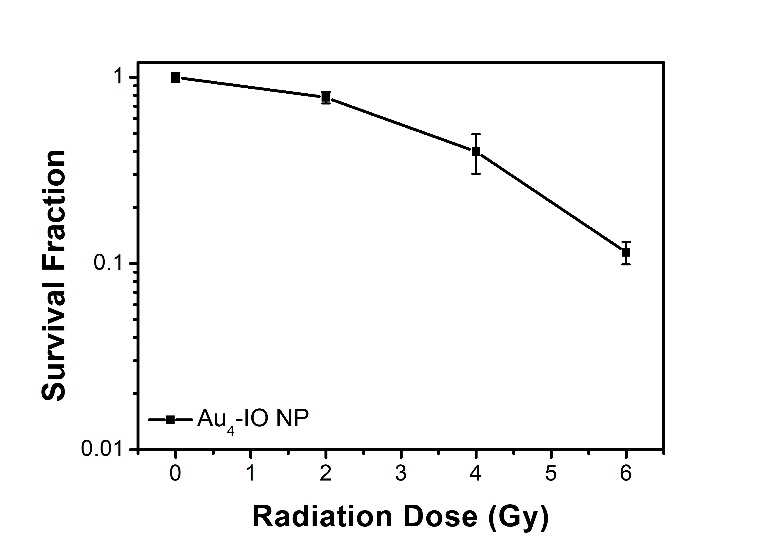


**Fig. S19** Survival curve of 4T1 cells received Au_4_-IO NP.


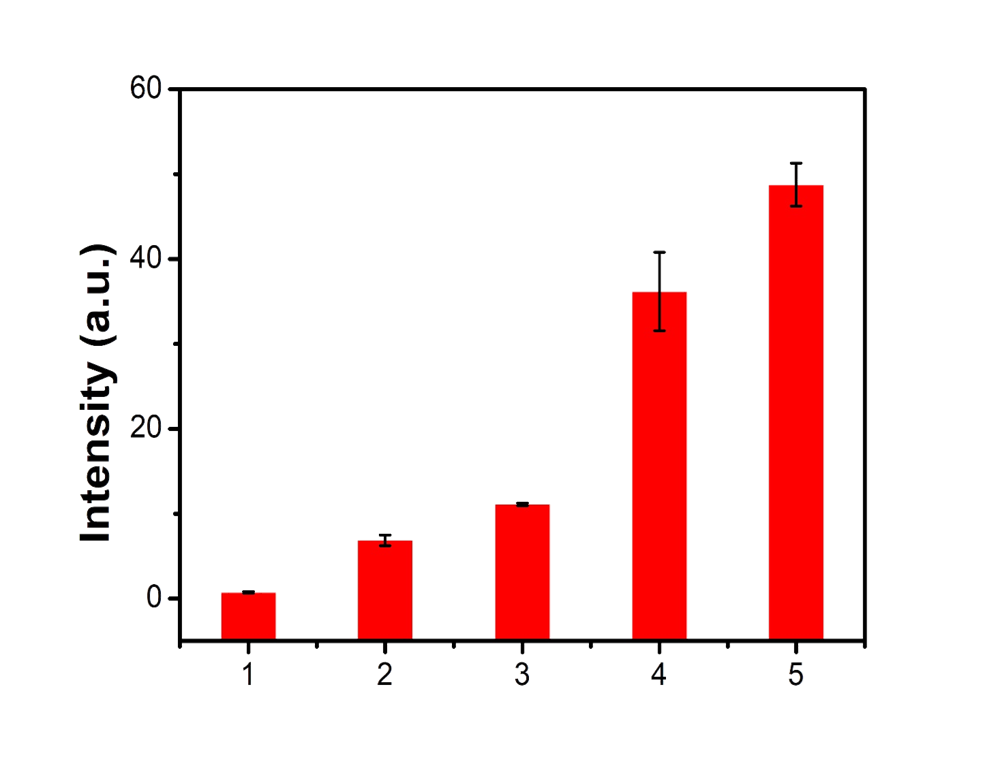


**Fig. S20** The fluorescence intensity of hydroxyl radical imaging of 4T1 cells (*λ_ex_*= 540 nm) at 6 h after administering different treatments. (1) Control, (2) Au_4_-IO NP-cRGD, (3) 4 Gy, (4) Au_4_-IO NP + 4 Gy, and (5) Au_4_-IO NP-cRGD + 4 Gy.


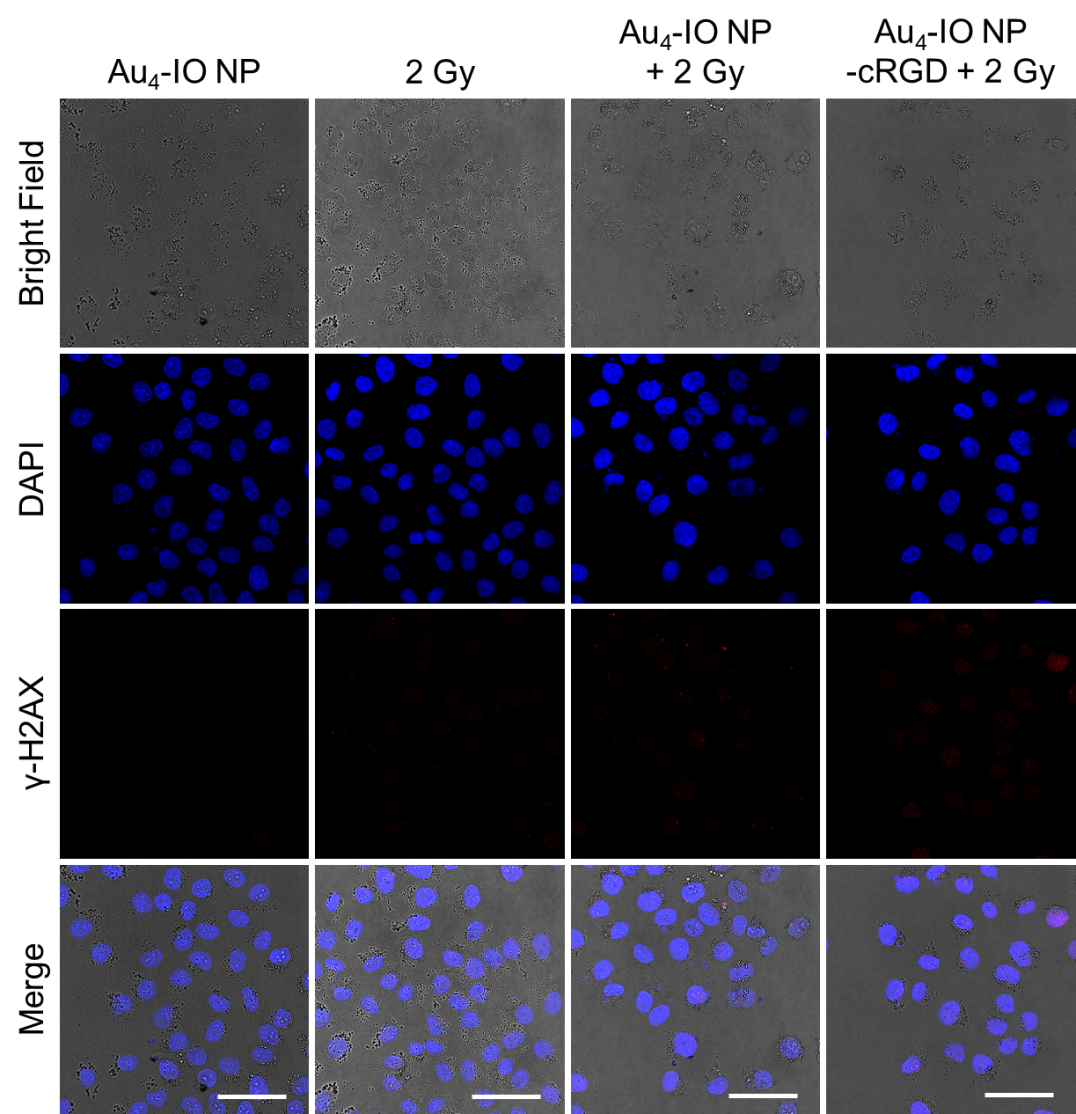


**Fig. S21** Confocal images of γ-H2AX expression in 4T1 cells after receiving various treatments. Scale bar: 50 μm.


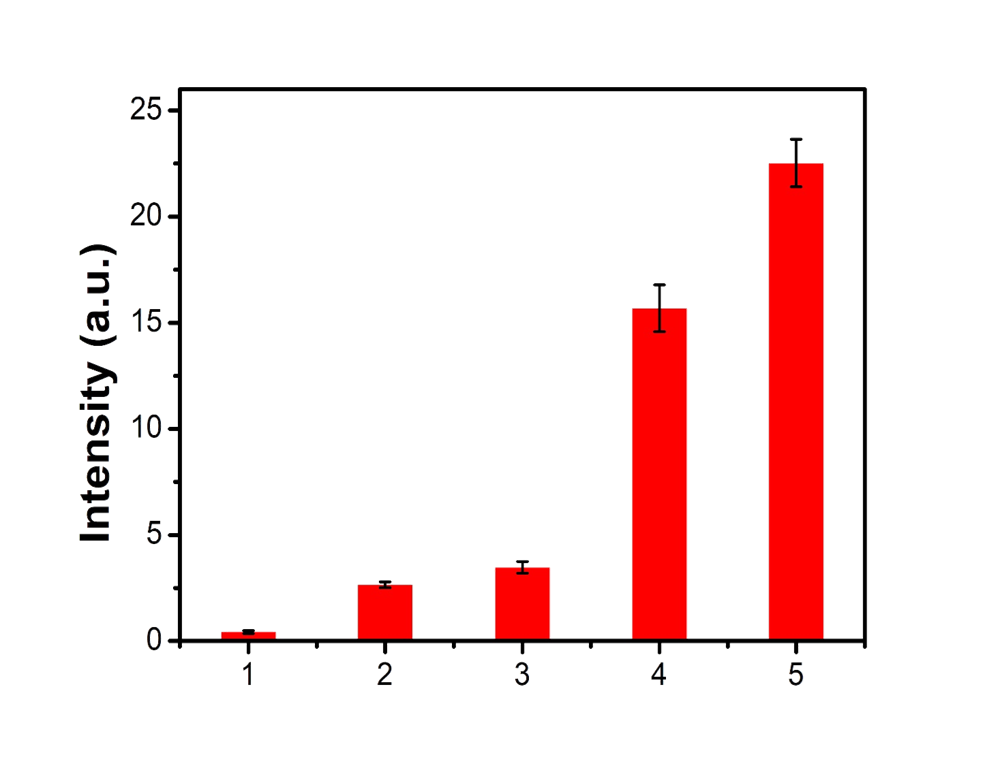


**Fig. S22** The fluorescence intensity of γ-H2AX imaging of 4T1 cells (*λ_ex_* = 647 nm) at 6 h after administering different treatments. (1) Control, (2) Au_4_-IO NP-cRGD, (3) 4 Gy, (4) Au_4_-IO NP + 4 Gy, and (5) Au_4_-IO NP-cRGD + 4 Gy.


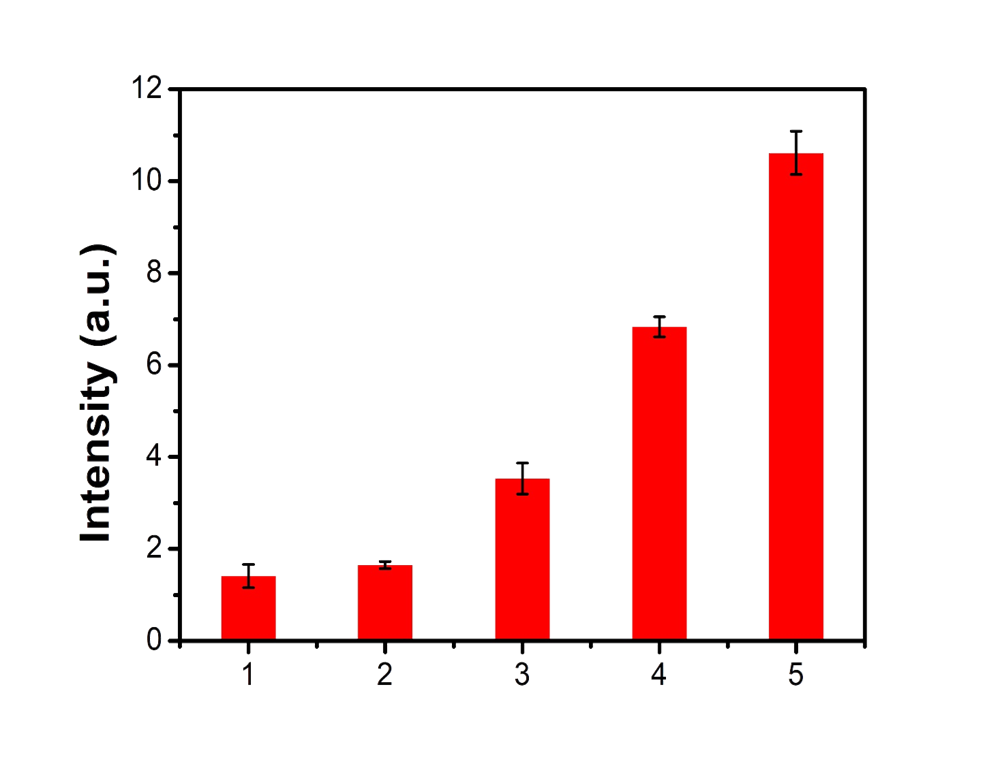


**Fig. S23** The fluorescence intensity of Caspase-3/7 imaging of 4T1 cells (*λ_ex_* = 488 nm) at 6 h after administering different treatments. (1) Control, (2) Au_4_-IO NP-cRGD, (3) 4 Gy, (4) Au_4_-IO NP + 4 Gy, and (5) Au_4_-IO NP-cRGD + 4 Gy.


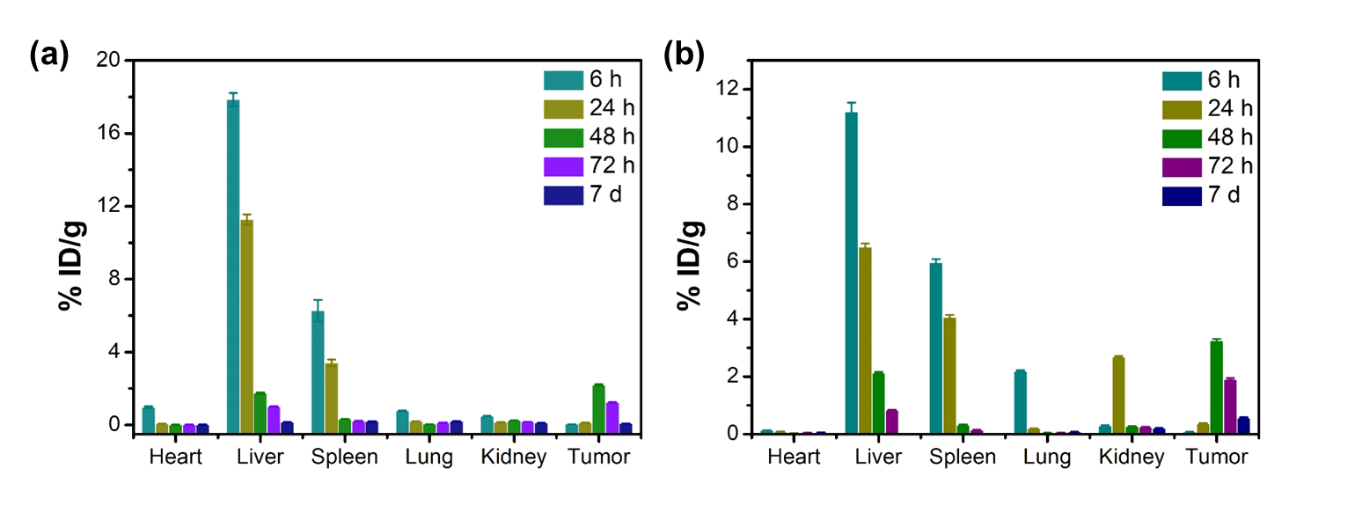


**Fig. S24** The biodistribution of (a) Au_4_-IO NP and (b) Au_4_-IO NP-cRGD at 6 h, 24 h, 48 h, 72 h, and 7 d after intravenous injection.


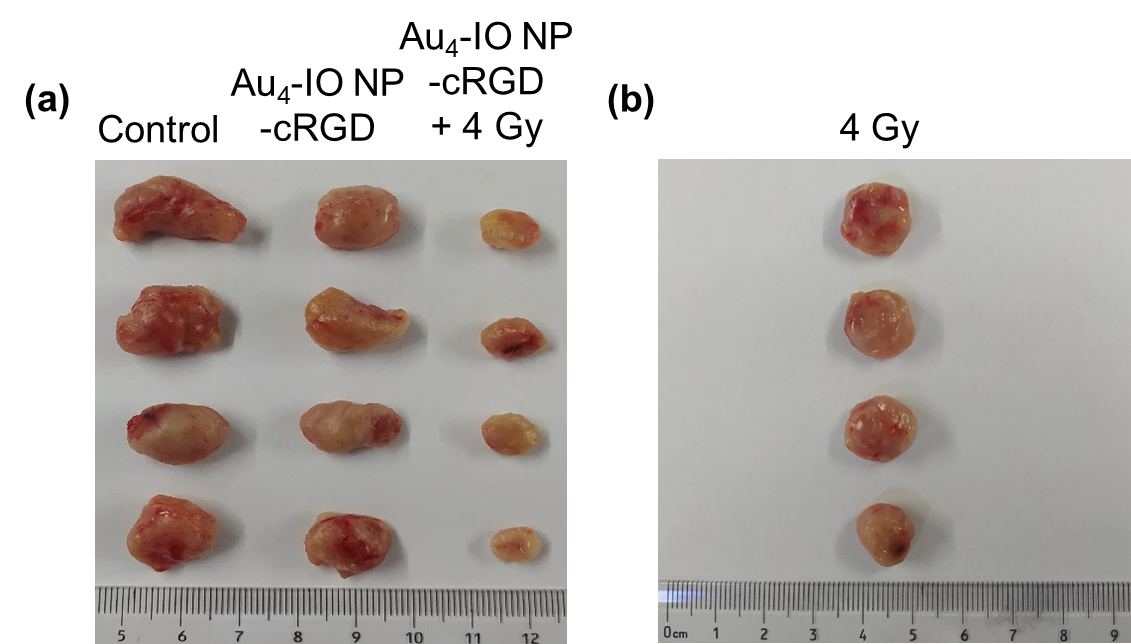


**Fig. S25** (a) Images of dissected tumors in control, Au_4_-IO NP-cRGD, and Au_4_-IO NP-cRGD + 4 Gy. (b) Images of dissected tumors in 4 Gy group.


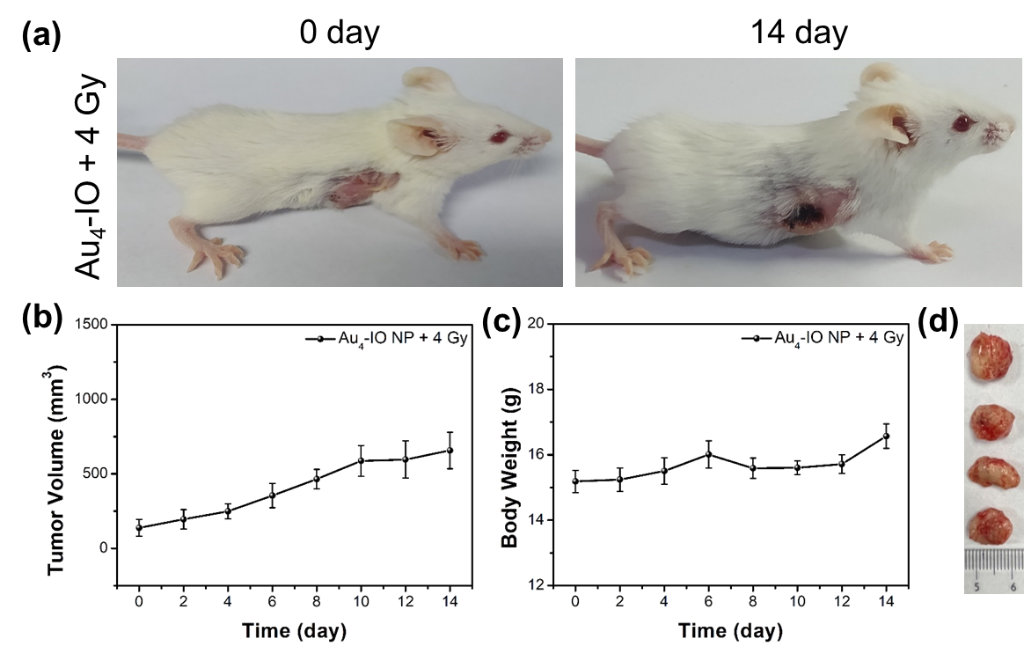


**Fig. S26** (a) Representative images of mice of Au_4_-IO NP + 4 Gy group at days 0 and 14. (b) Tumor volume curve of the mice. (c) Mouse body growth curve. (d) Image of dissected tumors in Au_4_-IO + 4 Gy.

**References**

1. Hooper TN, Butts CP, Green M, Haddow MF, McGrady JE, Russell CA, Synthesis, structure and reactivity of stable homoleptic gold(i) alkene cations. Chem Eur J. 2009;15:12196-200.
